# Deep phenotyping meets big data: the Geoscience and hEalth Cohort COnsortium (GECCO) data to enable exposome studies in The Netherlands

**DOI:** 10.1186/s12942-020-00235-z

**Published:** 2020-11-13

**Authors:** Jeroen Lakerveld, Alfred Wagtendonk, Ilonca Vaartjes, Derek Karssenberg, GECCO Consortium, Jeroen Lakerveld, Brenda Penninx, Joline Beulens, Erik Timmermans, Martijn Huisman, Alfred Wagtendonk, Sophia Kramer, Marieke van Wier, Dorret Boomsma, Gonneke Willemsen, Carlo Schuengel, Mirjam Oosterman, Karien Stronks, Derek Karssenberg, Roel Vermeulen, Ilonca Vaartjes, Annemarie Koster, Coen Stehouwer, Katja van den Hurk, Eric Koomen, Renée de Mutsert, Margreet ten Have, Monique Verschuren, Susan Picavet, Mariëlle Beenackers, Frank van Lenthe, Arfan Ikram, Vincent Jaddoe, Tineke Oldehinkel, Trynke de Jong, Saakje Mulder, Aafje Dotinga

**Affiliations:** 1 Department of Epidemiology and Data Science, Amsterdam Public Health Research Institute, Amsterdam UMC, VU University Amsterdam, De Boelelaan 1089a, 1081 HV Amsterdam, The Netherlands; 2 Global Geo Health Data Center, Utrecht University, Utrecht, The Netherlands; 3 Upstream Team, www.upstreamteam.nl, Amsterdam UMC, VU University Amsterdam, Amsterdam, The Netherlands; 4 Department of Epidemiology, UMC Utrecht, Div. Julius Centrum, Huispoststraat 6.131, 3508 GA Utrecht, The Netherlands; 5 Department of Physical Geography, Faculty of Geoscience, Utrecht University, Princetonlaan 8a, 3584 CB Utrecht, The Netherlands

**Keywords:** Exposome, Exposure, Upstream determinants, Big data, Environment, Cohorts, Non-communicable disease, Prevention, Data science

## Abstract

Environmental exposures are increasingly investigated as possible drivers of health behaviours and disease outcomes. So-called exposome studies that aim to identify and better understand the effects of exposures on behaviours and disease risk across the life course require high-quality environmental exposure data. The Netherlands has a great variety of environmental data available, including high spatial and often temporal resolution information on urban infrastructure, physico-chemical exposures, presence and availability of community services, and others. Until recently, these environmental data were scattered and measured at varying spatial scales, impeding linkage to individual-level (cohort) data as they were not operationalised as personal exposures, that is, the exposure to a certain environmental characteristic specific for a person. Within the Geoscience and hEalth Cohort COnsortium (GECCO) and with support of the Global Geo Health Data Center (GGHDC), a platform has been set up in The Netherlands where environmental variables are centralised, operationalised as personal exposures, and used to enrich 23 cohort studies and provided to researchers upon request. We here present and detail a series of personal exposure data sets that are available within GECCO to date, covering personal exposures of all residents of The Netherlands (currently about 17 M) over the full land surface of the country, and discuss challenges and opportunities for its use now and in the near future.

## Background

The exposome encompasses the life course exposures from lifestyle behaviours and from the environment [1]. The three broad exposome categories (i.e. ‘internal’, ‘specific external’ and ‘general external’) receive growing attention in epidemiological research with respect to its relationship with a variety of chronic diseases [2–4]. Environmental characteristics such as noise and air pollution, urban heat islands, walkability of neighbourhoods, living in an ‘obesogenic’ built environment may all influence disease risk directly, or indirectly via unhealthy dietary behaviours and physical inactivity. Given that many of the environmental factors are potentially modifiable, this provides a huge potential for prevention. Multidisciplinary and longitudinal research combining high quality individual-level data with environmental-level exposure data is urgently needed to identify and better understand their complex relations with each other and how they drive disease risk across the life course [5].

In The Netherlands, high quality and longitudinal data at the individual level as well as the environmental level exist. Various cohorts across The Netherlands contain longitudinal individual-level data on lifestyle behaviours and disease outcomes. The Netherlands also has a great variety of environmental data available, including high spatial and often temporal resolution information on urban infrastructure, physico-chemical exposures, presence and availability of community services, climate, and others. Until recently, these environmental data were scattered and available at varying spatial scales. Moreover, they were not operationalised as ‘personal exposures’ linkable to individual-level health data. Personal exposure encompasses the exposure to a certain environmental characteristic specific for a person. At population level it is not feasible to measure actual exposures ‘on the body’ by using sensors or other instruments. Rather, personal exposures can be estimated by averaging (or summing up, or otherwise aggregate) environmental attributes in a spatial and temporal context of an individual, mostly modelled over a specific distance zone (‘buffers’ or administrative neighbourhoods, or other geographic unit). Hereby it is assumed that people are more exposed to environmental attributes within a certain environment (e.g., home and/or work), depending on their socio-demographic characteristics and the exposure of interest. For instance, for older people, walkability of their neighbourhood would be assessed over an area relatively close to the home address as they generally have limited mobility, while noise pollution may be more relevant even at local address level and especially overnight.

Within the Geoscience and hEalth Cohort COnsortium (GECCO) and with support of the Global Geo Health Data Center (GGHDC), a platform has been provided for researchers to gain streamlined access to a wide range of personal exposure data. For this purpose, in a stepwise approach, environmental data are processed into personal environmental exposures, and environmental indices are developed such as walkability and drivability. These environmental exposures are available for researchers to use, and in the near future these data will be linked to the 631,000+ participants of 23 renowned and on-going large-scale Dutch cohorts that are currently affiliated to GECCO. This enables researchers from multiple disciplines to address a wide variety of research questions on environmental determinants of lifestyle behaviours and chronic disease risk.

GECCO started small, and has over the last years grown from enriching few cohorts with a good number of environmental exposures [6] to a solid infrastructure that contains 100+ environmental exposures at high resolution across various domains. While information on the individual-level cohort data are described elsewhere [7], we here present and detail a series of personal exposure data sets that are available within GECCO, covering exposures of all residents of The Netherlands (currently > 17 M) over the full land surface of The Netherlands, an area of about 33,680 km^2^. We also reflect on the challenges and opportunities for its expansion and use now and in the near future.

## Data collection, handling and quality control

### Prioritisation of data collection

Prior to the geodata collection and acquisition a literature scan was carried out in combination with a survey within the wider GECCO consortium. This was done to prioritize what environmental data to collect and process, so that foreseen users are better catered and a large variety of exposome studies could be carried out using the data. The literature scan included the assessment of key reviews on environmental determinants of chronic disease risk (e.g., [8–12]). The survey was thematically organised around 6 different spatial environment categories:

1. Physical activity environment
2. Transport/mobility environment
3. Environmental pollution
4. Food and retail environment
5. Socio-economic environment
6. Safety, aesthetics, air temperature.

For each of these six categories, respondents could indicate their interest for a number of pre-specified geo datasets (yes/no) or specific spatial indices (5–15 per category, 57 in total), and an open field was added to indicate other specific interests and suggestions. A total of 73 respondents from over 10 different GECCO-affiliated organisations completed the survey. The survey results showed that virtually each listed dataset was of interest to at least a few respondents, and approximately a third of the proposed datasets generated the interest of the majority of the consortium. Data sets with high level of detail generally gained more interest, e.g. the availability of alcohol and tobacco in the food retail environment, while at the same time there was also ample interest for aggregated data (factors combined in a single construct, such as walkability) in larger spatial units. Together these results implied the desire for a large variety of personal exposure data in terms of thematic and spatial detail and temporal ranges.

**Fig. 1:**
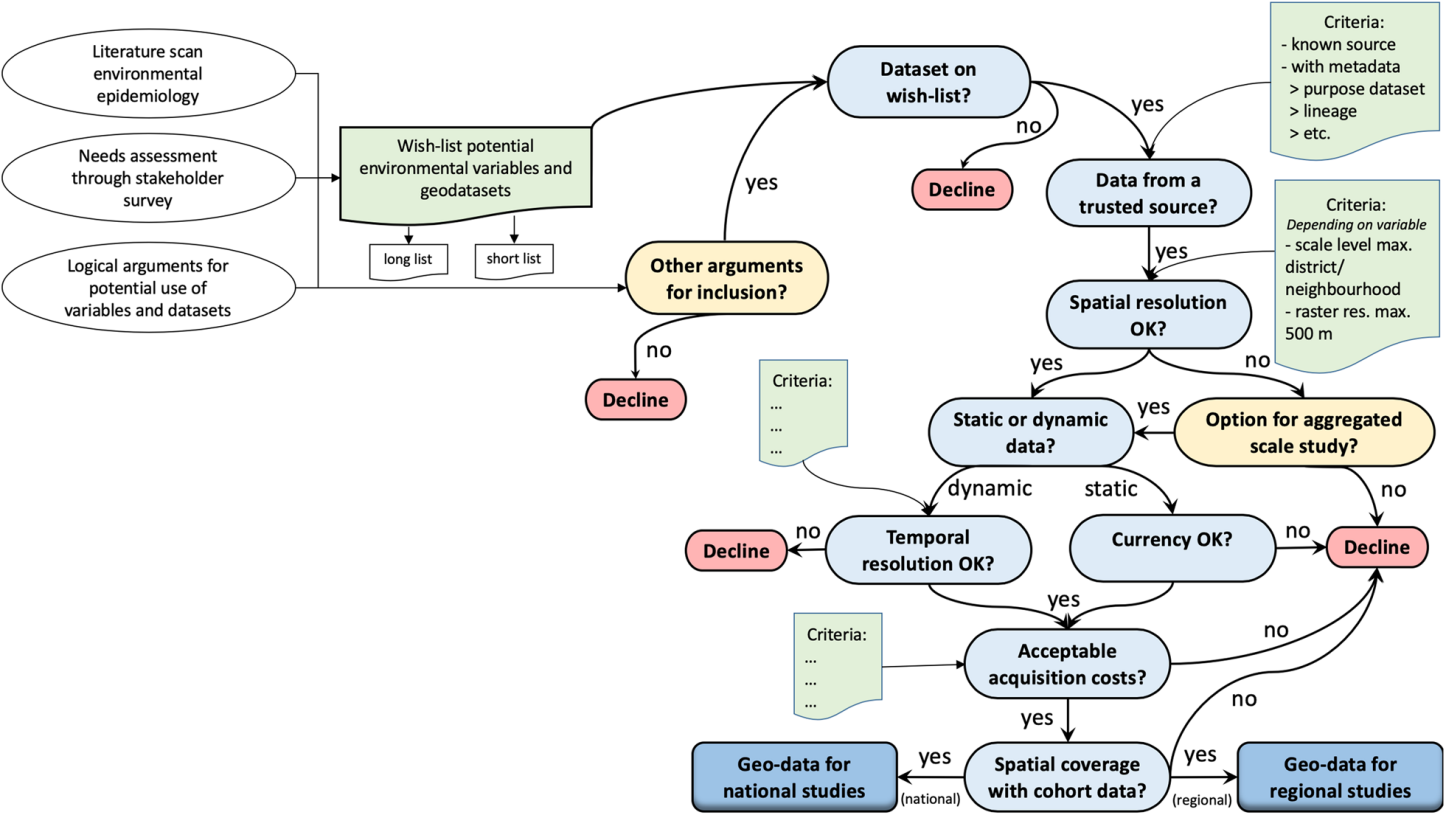
Decision tree with the different criteria used and decisions taken during the selection of geodata and the production of environmental variables for GECCO

Next to the literature scan and the survey, the prioritisation of our data collection was informed by the following factors (see also Fig. 1):

- Data quality and trustworthiness of data source (e.g. is it a known data source with metadata on purpose, quality and other relevant characteristics)
- Minimum spatial resolution (e.g. neighbourhood level for administrative data and 500-m resolution for raster data)
- Temporal resolution (e.g. for highly dynamic data such as average temperatures much higher resolutions—often daily or monthly—are necessary than for semi-static data such as road infrastructure, for which 5-yearly updates are sufficient)
- Thematic resolution (e.g. can built-up area in a land use dataset be divided in specific classes such as residential area, office area, industrial area, retail area, social-cultural services, etc.)
- Costs and use restrictions (e.g. cost of a dataset can be too high in relation to available budget and the relevance of the dataset; the use of the data is only allowed by the data-owner for the research project the data was acquired for).

In general, these criteria were pragmatically applied and meant we gave priority to affordable or free datasets of higher quality, with a high spatial, temporal and thematic resolution for data themes that were of sufficient interest for our targeted user community.

## Spatial data sources

Professional open geospatial data of The Netherlands with minimal quality standards accompanied with a metadata description can be found via the national clearing house [13] and/or the national public geodata platform PDOK [14] together with a map and download service for location data in tabulated form or data in geographic information systems (GIS) formats. Examples of such datasets concern altitude data, topographical key registrations, cadastral maps, protected areas, national cycling and walking routes, aerial photography and so on.

Special clearing houses also exist for more thematic spatial datasets such as the National Data Warehouse for Traffic Information (NDW) [15], open government data [16], open education data [17] or the Environmental Health Atlas [18]. Examples of data that were found this way are essential geodata sources such as topographical data by the Dutch cadastre, neighbourhood characteristics and land use data by the Statistics Netherlands (CBS) and health or noise data via the National Institute for Health and Environment (RIVM).

While these sources account for a large share of the available geodata, still a considerable share of geodata, both open geospatial and commercial geodata, is available only via specific spatial data sections of professional organisations themselves, such as certain scientific data produced by universities, research institutions and geodata companies. Examples are air pollution datasets on address level produced by the European Study of Cohorts for Air Pollution Effects (ESCAPE) [19], poverty maps on postcode 4 level produced by The Netherlands Institute for Social Research (SCP), sport accommodation address locations by the Mulier institute, or (commercial) retail address locations by Locatus [20].

Another category of (semi)professional data can be found in the form of voluntary collected geodata, such as road data and points of interest in the OpenStreetMap project. Sometimes recent data of a certain theme can be found via the national clearing house, but older historical data only via the data providers themselves or via specialised research data archives such as Data Archiving and Networked Services (DANS). On top of this, national branches of commercial geo software companies such as ESRI offer free geodata services in the form of pre-processed national datasets in GIS ready formats for example for the key registries on topography, buildings and addresses [21].

Besides the data on a national scale, large quantities of geodata are available on regional to local scales for which e.g. provinces and municipalities can be excellent sources. Subnational datasets are collected by the GECCO project on specific request.

Finally, relevant geodatasets exist that were not (yet) published online, except by mentioning in a report or research paper. To acquire these types of datasets, specific requests to the data owners were made.

### Processing steps from geodata to personal exposure variables

Selected datasets downloaded from FTP-sites and data repositories concerned spatial data in different kind of file formats and were transformed into standard GIS vector and raster formats and where necessary projected or re-projected to the Dutch coordinate system (*Rijksdriehoekstelsel*).

To produce a basic set of spatial variables the geodatasets were processed further using common spatial operations, such as spatial selections/extractions (e.g. from European to national extent), spatial aggregations to summarize data (e.g. point/line vector data or high resolution raster data) to administrative units, joining of attribute data to administrative units (e.g. data national statistics office to neighbourhoods or PC4 areas), merge or dissolve operations, buffering, reclassifications of thematic data, as well as data enrichment using auxiliary data. An example of the latter operation was the preparation of a land use mix variable where we ‘enriched’ the national land use data by disaggregating the land use class ‘commercial areas’ to two separate classes ‘industrial/manufacturing area’ and ‘office space’ by using detailed polygon data on the utilization of buildings in the national key register on addresses and buildings (BAG). More specific spatial variables with different personal exposure areas were constructed using spatial functions such as neighbourhood analysis, kernel density, zonal statistics and by making specific combinations of variables.

The next procedural step was to convert the environmental data to personal exposures, which is the exposure of individuals in their so-called spatial context or exposure area. This step involves the statistical aggregation (e.g. count, average) of environmental variables over areas surrounding each of the residential locations in The Netherlands. In a number of cases there was no need to aggregate values over a spatial context, for example when the exposure was mostly relevant for the location of residence itself, such as exposure to noise during the night. In those cases, the value of the environmental attribute at the location of the front door or at the centre of the building was directly assigned to that residential location.

**Fig. 2:**
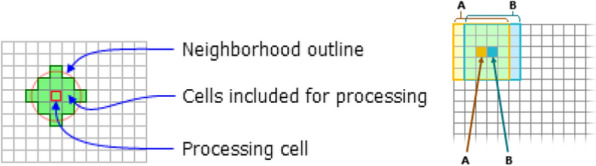
Concept of moving window/neighbourhood analysis in GIS. For explanation refer to main text

More often, however, epidemiologists are interested in a statistical summary of data within the exposure zone around an address location. This can be the administrative neighbourhood or 4/6 digit postal code areas in which the address is located or one or more (usually) circular shaped exposure radii of any distance usually between 100 and 2000 m. In that case the radius distance can depend on the expected activity space for e.g. walking, cycling or driving of a certain target group. Alternatively, the exposure area can have different forms, including irregular forms, e.g. on the basis of calculated travel distances over the roads (e.g. the area reached within 5 min walking distance) or the exposure area is not centred around an address location but around a certain destination e.g. to determine which addresses fall inside the service area of a certain school or health service. Furthermore, in some cases we have weighted also the distance to individual features within an exposure zone by applying kernel density analysis. Kernel density analyses take distance to—for example food retailers—into account as well as density, by assigning more weight to more nearby features than to features further away according to a certain distance function and, this way, produce a continuous density surface. For example, the standard kernel density function in the ArcGIS software uses the ‘quartic kernel function’ described in Silverman [22], and works by fitting a smoothly curved surface over each feature point within the exposure zone, with a surface value diminishing from the central point to a value of zero at the search radius distance. The kernel density at each output raster cell is subsequently calculated by adding all the values of kernel surfaces where they overlay the raster cell centre. Figure 4 gives an example in which this kernel density function was used to produce distance-based kernel densities of supermarket access within a 1000-m radius. A relatively simple example of personal exposure assessment from environmental data in a raster format at high resolution (25 m) is shown in Fig. 2. The left panel shows a particular processing cell containing a residential location and a circular exposure area over which the environmental attribute is aggregated. On the right, it is shown how this calculation is done for each processing cell by moving the exposure area, here shown as a square box. Point, vector, as well as raster data can be input for such an analysis and the result can be linked to cohort data on address level or cohort data on lower scale levels.

The final step was to produce personal exposures for different exposure areas in table format suitable to link to individual-level (cohort) health data on either address, 6-digit postal code (PC6), 4-digit postal code (PC4), neighbourhood or in some cases district or municipality levels.

For all collected geodata that were processed into a final GECCO product, a metadata-sheet was created containing all the relevant characteristics of the data, guided by general principles and standard metadata requirements serving discovery, evaluation and use of spatial data (see Annexes S1-S3 for examples). ArcGIS (version 10.6 or higher) from ESRI with the Spatial Analyst extension was used for most of the spatial operations in combination with QGIS (version 3.0 or higher) for some specific operations. Several parts of the variable production process were automated using Python scripts with the Python site package ‘ArcPy’ for utilizing spatial functions available in ArcGIS.

Despite the use of high-end computers with high processing speed and large working memory, very large repetitive database operations could not be executed in acceptable processing times. For producing multiple exposure variables on the address level by extracting and joining geographic data to over 9 million address coordinates, we therefore used process scripts written for execution in a specific spatially aware software called Geo Data and Model Software (GeoDMS). The GeoDMS is a calculating engine that was specifically designed to process, calculate and visualize large (geographic) datasets. All datasets are stored on secured university network servers, which are rigorously protected and regularly being backed-up.

**Fig. 3:**
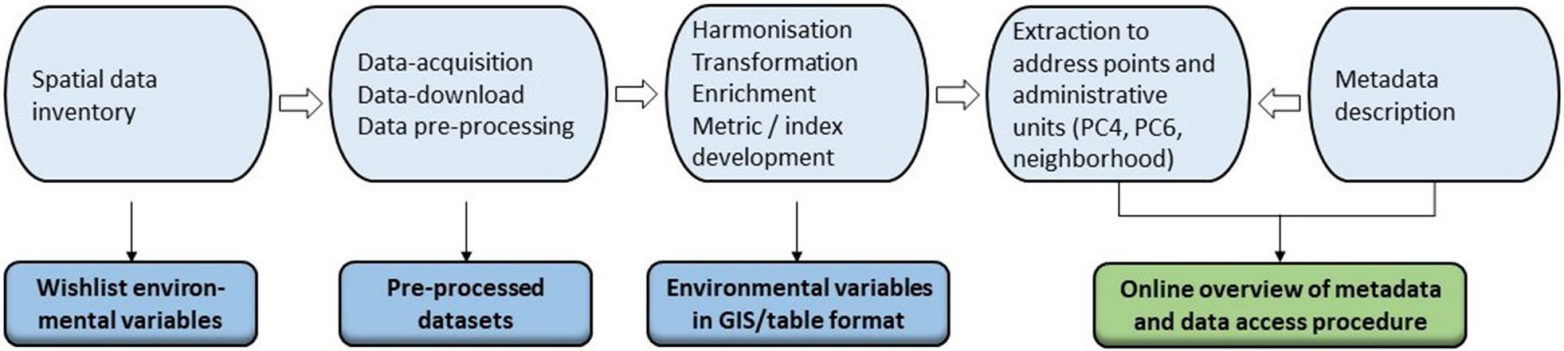
Overview of different steps and products in the process from original data to environmental exposure variable

Figure 3 provides an overview of the different steps and products in the process from original source data to environmental exposure variable.

**Fig. 4:**
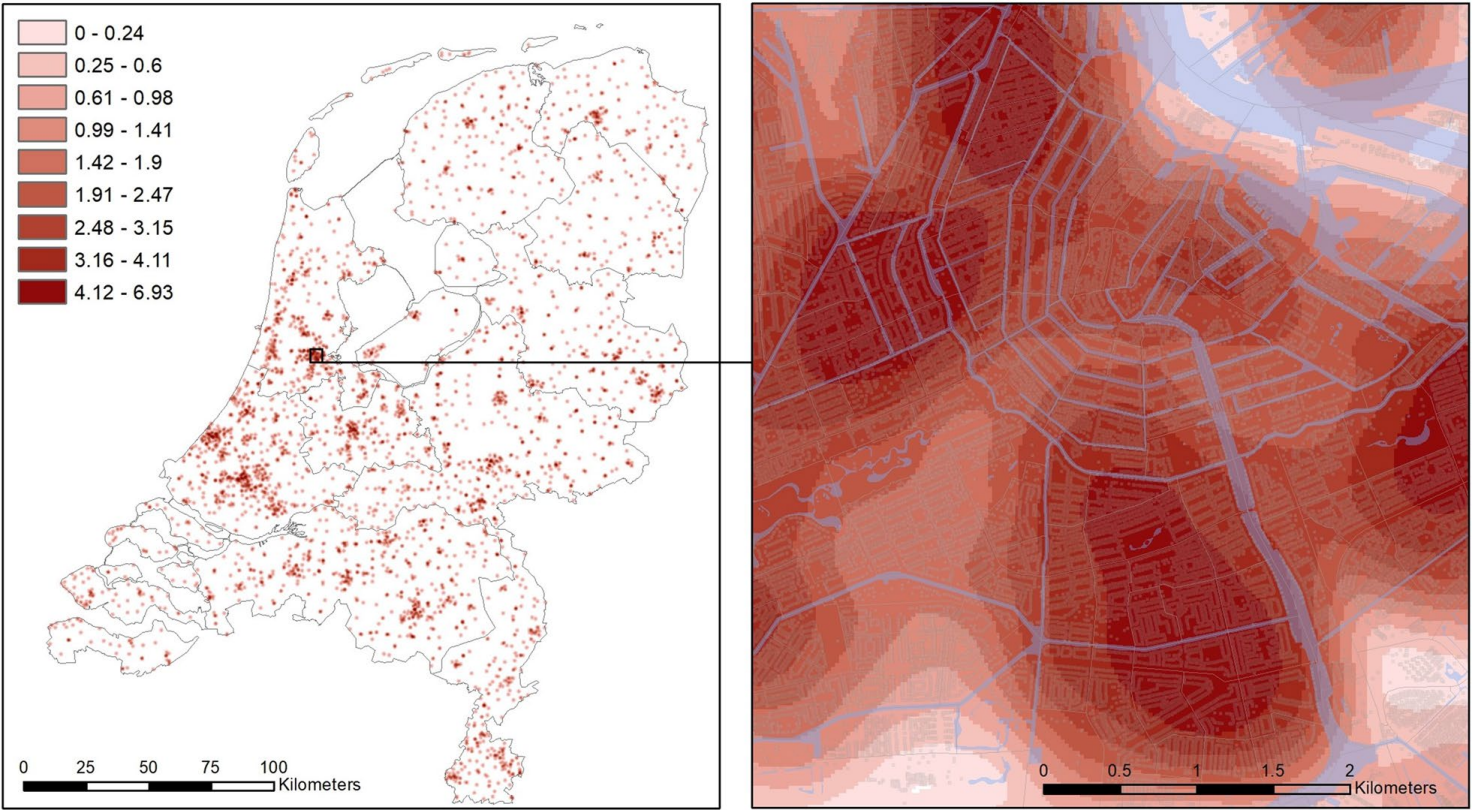
Map example showing the kernel density (in average number of supermarkets per km^2^) of supermarket access within a 1000-m radius for the Netherlands (left) and the Amsterdam region (right) in 2008, where dark red indicates higher access

**Fig. 5:**
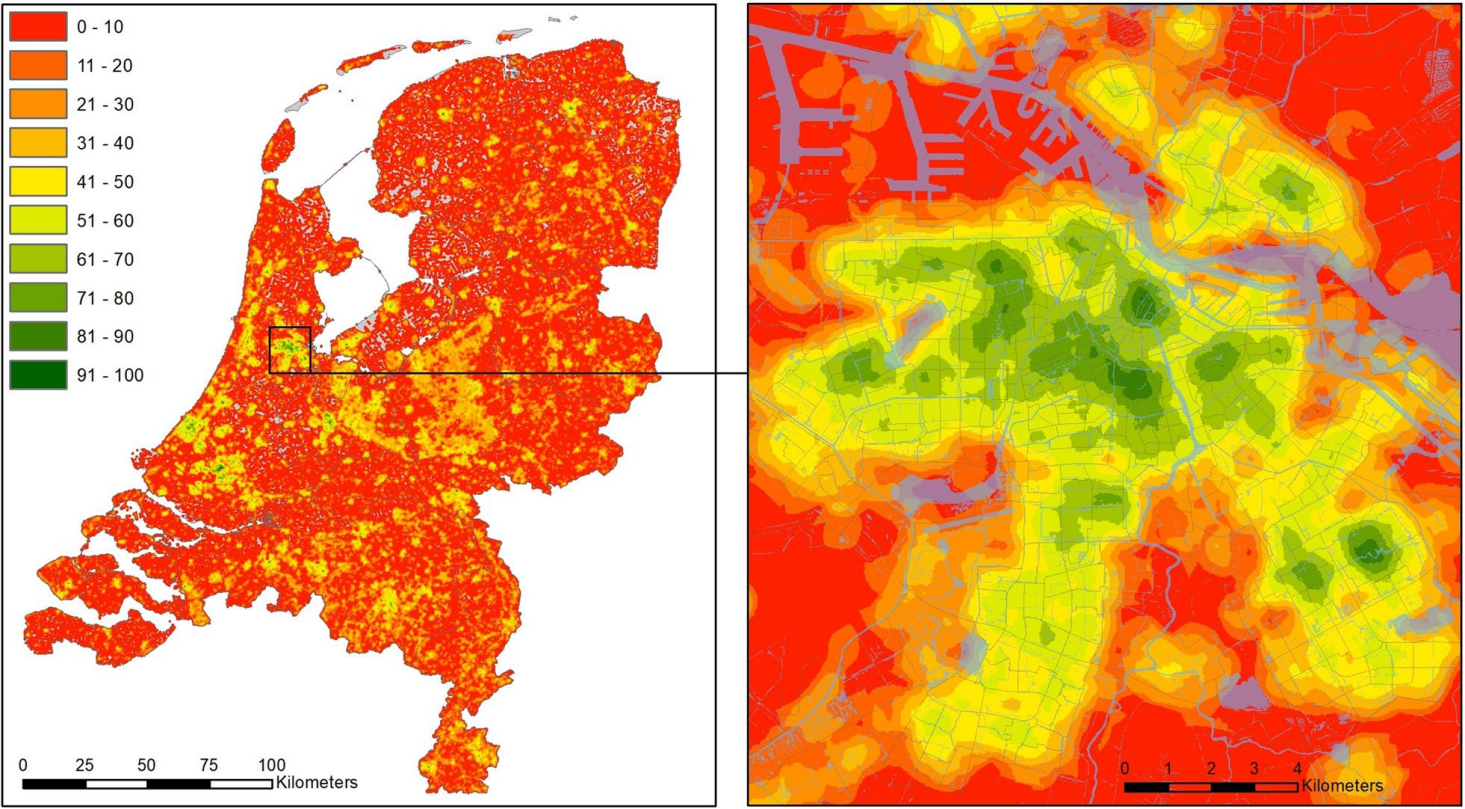
Map example showing the walkability scores (range 0–100) for a 500-m exposure area of The Netherlands (left) and the Amsterdam region (right) in 2015, where green indicates higher walkability

Figures 4 and 5 below provide map examples of respectively a kernel density based environmental exposure variable and a compound index variable based on six sub variables.

### Geographic issues and quality aspects

During the various transformation processes known geographic issues were encountered that needed to be addressed. A common issue is for example the Modifiable Areal Unit Problem (MAUP). The MAUP leads to one of the well-known challenges in spatial epidemiological research and other population health studies [23, 24] and occurs when e.g. point-based measures of spatial phenomena are aggregated into administrative units in which summary values (e.g., totals, rates, proportions, densities) are influenced by both the shape and scale of the aggregation unit. Fully resolving this issue is currently not feasible, but to address and reduce this specific problem, we calculated point density kernels prior to aggregating data to neighbourhoods. Doing this accomplished that distance weighted environment information around each data-point was gathered and summed up in a regular spaced raster and subsequently averaged over the corresponding neighbourhoods. In effect this meant that also cross-border environment information was incorporated into the data aggregations of each neighbourhood. This procedure to reduce the MAUP is, however, only necessary when it cannot be avoided to aggregate data to administrative units such as neighborhoods, e.g. when health cohort data is only available on a certain administrative scale level. More sophisticated methods have been developed to deal with MAUP and related geographic issues, such as Bayesian hierarchical models and Geographically Weighted Regression with a focus on local spatial regression rather than global regression [25], but in general we recommend to avoid any aggregation of available geographic data to administrative units and work only with uniform exposure units, such as the circular exposure radii that are often used in the studies related to GECCO. Furthermore, as suggested by Fecht et al. [26] we recommend to look for a spatial unit of analysis that reflects as much as possible the expected geographical scale of interaction between the spatial determinants and the health outcomes. In case data aggregation to administrative units cannot be avoided we recommend to carry out the proposed method for reducing MAUP effects and additional sensitivity analysis with different spatial scales for the assessment of remaining MAUP effects on the results. Another known geographic issue is the Uncertain Geographic Context Problem, relating to the chosen area and time of exposures—which might not accurately represent the actual area, time and/or duration that exert contextual influences on the health behaviours or health outcome under study [27, 28]. Ideally, addressing this issue would mean that more appropriate contextual units would have to be de-lineated. That means that these units have to be based on people’s actual or potential (often multiple) activity spaces [28], and determining these, e.g. with GPS based activity survey data. Unfortunately, this is an unattainable objective for most studies. In any case, the decision on what specific area of exposure and time of exposure to use will be specific to the research population and question under study, as well as the available survey- or cohort data that will be linked. Within GECCO, most exposures are therefore calculated for different points in time and a range of area types and sizes, as detailed further down. In addition, as recommended, we and others encourage researchers to develop an adequate theoretical model for taking spatial and temporal contextual uncertainties into account, to do sensitivity analyses with other area sizes, and choose exposures that are measured with the narrowest possible time gap [28–30].

To make a proper evaluation possible of the fitness for purpose of the produced environmental variables, we provided relevant metadata on the primary (original) data sources, as well as metadata on the details and applied processes towards the secondary (derived) geodata and environmental variables. The majority of the original data comes from formal national bodies such as the Dutch statistics office (CBS), Dutch environmental assessment agency (PBL) or the Dutch cadastre and are usually subject to internal quality control procedures and provided and catalogued with detailed metadata based on international standards such as ISO (e.g. the Dutch metadata profile ISO 19115 for geographical data and/or the European metadata standard INSPIRE for spatial data).

However, more specific geodatasets such as the national dataset sport accommodations (Mulier sport-research institute), the public transport stops dataset (Groningen University/NDOV), or the Locatus retail data, do not always contain standardized metadata descriptions. Therefore, besides providing available metadata as much as possible on both primary data and secondary data, we carried out random verification-checks of areas that are familiar to us, before delivering requested environmental variables to researchers. For the Locatus retail-data we carried out a separate verification study [31].

### List of environmental exposures

Although the final products of the GECCO project are environmental variables in table format, the produced intermediate geo datasets have an essential role in the project. Any desired variable that is spatially different from the standard set of produced variables (e.g. updated neighbourhood borders, larger exposure radius) needs to be reproduced on the basis of a pre-existing geodata set.

In depicting our list of environmental exposures here, we chose to distinguish 6 categories of exposures and classify geodatasets and derived environmental variables with their different exposure zones according to these environments (see Table 1). Some of the datasets and/or derived environmental variables could be allocated to other health environments as well, e.g. neighbourhood data contains a clear administrative-demographic component as well as a socio-economic component that could also be classified to the social-cultural component.

**Table 1:** Availability of personal exposure variables and data sources

Exposure category	Environmental variable(s)	Period	Exposure zone(s) A(r) = address radius (m) Ac = address coordinates NB = neighbourhood P4 = 4-digit postal code P6 = 6-digit postal code	Geodata source	Remarks
1. Physical activity environment (infrastructure and land use deter-mining the way the surroundings can be accessed and used)	Altitude in centimetres	2000–2018	Ac, NB, P4, P6	AHN.nl—cooperation of provinces, central government and water boards	The altitude map of the Netherlands is a laser altimetry product in raster format available on different horizontal scales levels
	-25 m. resolution (AHN1)	2000 (ca.)			
	-5 m. resolution (AHN2)	2010 (ca.)			
	-50 cm. resolution (AHN3)	2018 (ca.)			
	Bicycle path density	2019	NB	Basic topography register system (BRT—TOP10—Cadastre, 2019) with point and line layers of roads, railways, junctions, ramps and exits, bridges, tunnels, cycle lanes, footpaths, etc	Topographic cycle path line data joined with data ‘Landelijk fietsplatform
	Road density	2015	NB		The (car)road density is derived from the dataset TOP10 NL 2015 (line feature layer WEGDEEL_HARTLIJN)
	Street connectivity	1989 1993 2001 2003 2012 2015 2019	A_150,250,350,500,750,_ 1000,1650,2000	Key register Large-scale Topography (BGT—Cadastre) including among others polygon layers of separate bicycle lanes and sideways	Connectivity of the street network, represented by the ratio between the number of true intersections (three or more legs) to the size of the selected area
			NB, P4, P6		
	Sidewalk density	1989 1993 1996 2000 2003 2008 2012 2015 2019	A_150,250,350,500,750,_		Density of sidewalk polygon area calculated as Z-scores. Years before 2015 are constructed using auxiliary data
			_1000,1650,2000_		
			NB, P4, P6		
	Land use	1989 1993 1996 2000 2003 2006 20082020 2012 2015	Ac, NB, P4, P6	Land use—Statistics Netherlands (CBS) based on a.o. TOP10 and aerial photography. Classification in 9 main land use classes and ca. 40 subclasses	Land use concerns generalized data. Classification changes occur between 1993 and 1996
	Land use mix/ entropy index	1989 1993 1996 2000 2003 2006 2008 2010 2012 2015	A_150,250,350,500,750, 1000,1650,2000_ NB, P4, P6	Land use—Statistics Netherlands (CBS)	The land use mix is calculated as Z-scores and indicates the heterogeneity of five specific land use classes
	Land use classes				
	1-residential				
	2-commercial				
	3-social-cultural services				
	4-offices/ public services				
	5-greenspace/ recreation				
	Green space density	1989 1993 1996 2000 2003 2006 2008 2010 2012 2015	A_150,250,350,500,750, 1000,1650,2000_ NB, P4, P6	Land use—Statistics Netherlands (CBS)	Greenspace density calculated as Z-scores. Greenspace includes public gardens, parks, forests and graveyards
	Green space (10 m. res.)	2017	NB, P4, P6	Institute for Public Health and the Environment (RIVM)/ Atlas Leefomgeving (ALO)	Combination of different datasets related to green space derived from the AHN2 and AHN3 files, the BAG buildings and the Infrared aerial photo (CIR file, resolution of 0.25 m)
	-% Trees				
	-Tree height classes				
	-% Shrubs				
	-% Low vegetation				
	Sport accommodation density (indoor and outdoor)	2017	NB	Databestand SportAanbod (DSA) Mulier instituut	Accommodation density is calculated from a national dataset with xy coordinates from ca. 22.000 sport accommodations managed by the Mulier institute
	Base topography—TOP10 BRT—(a.o. roads, tracks, water, terrain, furnishing elements)	2003 2005 2010 2011 2012 2013 2015 2019	NB, P4, P6	Basic topography register system (BRT—TOP10—Cadastre	
	Key register large-scale Topography—BGT (point, line and polygon layers of topographical objects)	2012–2020 continuous	NB, P4, P6	Key register large-scale Topography—BGT—Cadastre	Application scale 1:500–1:5.000
	Walkability index	1989 1993 1996 2000 2003 2006 2008 2010 2012 2015	A_150,250,350,500,750, 1000,1650,2000_ NB, P4, P6	GECCO project based on land use and population Statistics Netherlands (CBS) and basic/ large scale topography Cadastre Netherlands	Walkability is calculated by summing the z-scores of its six components and normalizing the results to values between 0 and 100
	Composite score based on six components:				
	1) Population density				
	2) Density of retail and service destinations				
	3) Land-use mix				
	4) Street connectivity				
	5) Green space				
	6) Side walk density				
	Bicycle and walking network including cycling and walking routes, networks and transport nodes	2019 continuous	NB, P4	Derived from TOP10 NL road data by Landelijk Fietsplatform and Wandelnet	Vector line data
2. Transport/mobility environment	Parking spaces (public street parking spaces, private residential places and paid/ unpaid parking garages and car parks)	2019 (park spaces BAG 2015)	NB	Derived from dataset ‘Parking places’ Cadastre/ RDW (Netherlands Vehicle Authority). Combines vector point and polygon data from BGT, TOP10, BAG and RDW on scales 1:2.500–1:10.000	Statistical summaries have been made for the neighbourhood borders of 2016. The BAG data for private built-up parking spaces concerns the year 2015, the other data concerns 2019
	-Number of parking places				
	-Park space density in				
	number of parking places per household				
	-Number of parking places per hectare				
	-Park space ratio as				
	-Number of cars/ number of parking places				
	Public transport stop density (bus, ferry, metro, taxi and tram stops)	2018 (updated from 2015)	NB	Geodienst Rijksuniversiteit Groningen/ databank Nationale Data Openbaar Vervoer (NDOV)	Kernel point densities (1000-m search radius) of public transport stops are calculated to overcome MAUP neighbourhood effect
	Railway stations	2019	A(r), NB, P4, P6	Esri Netherlands Datasets	On the basis of this dataset several distance and density based exposure variables can be derived on request
3. Environmental pollution (pollution/ nuisance in surroundings, air, soil or water, measured, modeled and/or perceived)	Traffic noise—daily mean (mixed road, rail and air) in Lden	2000 2004, 2005 2007 2008	Ac, P4, P6	PBL Netherlands Environmental Assessment Agency	Modelled data with Empara noise tool with 25 × 25 m resolution on mixed traffic noise in dB
	Traffic noise—daily mean (road only) in Lden	2000 2004 2007 2008 2010 2011	Ac, P4, P6	PBL Netherlands Environmental Assessment Agency	Modelled data with Empara noise tool with 25 × 25 m resolution on road noise in dB. Several factors are accounted including traffic intensity, road types and sound barriers
	Traffic noise— national roads (high ways)	2006 2011 2016	Ac, P4, P6	Dep. of Waterways and Public Works (Min. of IenW)	
	Airport noise Schiphol	2016	Ac, P6	Ministry of Infrastruc-ture and Water Management (IenW)	Separate data available for day and night (noise in Lden)
	Air pollution < 25 m resolution modelled annual average of min., max. and mean values	2009	Ac, P4, P6	Institute of Risk Assessment Sciences (IRAS)/ European Study of Cohort for Air Pollution Effects (ESCAPE)	Annual average outdoor pollution concentrations modelled/ interpolated with measurement data, traffic data and the physical environment. See online mapviewer
	-Particulate matter (PM_2.5_)				
	-PM 2.5 absorbance				
	-Particulate matter (PM_10_)				
	-Particulate matter (PM_coarse_)				
	-Nitrogen dioxide (NO_2_)				
	-Nitrogen oxide (NO_x_)				
	Air pollution 25 m. resolution modelled annual average -Particulate matter (PM_2.5_) -Particulate matter (PM_10_) -Nitrogen dioxide (NO_2_) -Soot (EC)	2013 2014 2015 2016 2017 (NO_2_ not for 2013)	Ac, NB, P4, P6	Institute for Public Health and the Environment (RIVM)	Annual average outdoor pollution concentrations based on a combination of model calculations and measurements from official measurement locations. SOOT (EC) maps indicative only
	Air pollution 1 km resolution modelled annual average	1995-2018 Yearly	Ac, NB, P4, P6	Institute for Public Health and the Environment (RIVM)	Modelled future concentra-tions are available for all variables for 2020, 2025 and 2030, apart for C_6_H_6_ and CO
	-Benzene (C_6_H_6_)	2011–2018			
	-Carbon monoxide (CO)	2011–2018			
	-Carbon monoxide p98 (CO)	2011–2018			
	-Particulate matter (PM_2.5_)	2017–2018			
	-Particulate matter (PM_10_)	1995–2018			
	-Ammonia (NH_3_)	2011–2018			
	-Nitrogen dioxide (NO_2)_	1995–2018			
	-Nitrogen oxide (NO_x_)	2011–2018			
	-Ozone (O_3_)	2011–2018			
	-Soot (EC)	2011–2018			
	-Sulphur dioxide (SO_2_)	2011–2018			
4. Food and retail environment	Food environment healthiness-index (other variables derived from Locatus point data on request)	2016 (other years on request)	NB (on the basis of this dataset several distance and density based exposure variables can be derived on request)	Retail point coordinate data LOCATUS (2004–2020)	Index score (food environment healthiness index) between − 5 and + 5 according to FEHI score as described elsewhere [32]. Data is aggregated to neighbourhoods using point density kernels to prevent MAUP issue
5. Socio-economic environment (administrative divisions, key demography, social and economic parameters and cultural amenities)	Neighbourhood statistics	Two-yearly 1995–2001 One-yearly 2002–2019	Ac, NB	‘Wijk- en buurtkaarten’ Statistics Netherlands (CBS)	The Dutch statistical office (CBS), records a range of demographic variables per neighbourhood Neighbourhood borders/divisions can change over the years and also the recorded variables can change over the years
	-Demographics (age classes, sex, mortality, etc.) -Population density				
	-Provenance-Urbanization				
	-Housing stock-Living (rent, ownership, residence types, etc.)-Energy consumption (gas/ electricity)-Education-Labour-Income				
	-Crime-Social security -Businesses-Motor vehicles -Land use				
	-Amenities (average distance to specific facilities and average number of specific facilities within a radius around addresses in a neighbourhood)-Overlapping PC4 area-Area land/water				
	Buildings/addresses (BAG)	2011–2020 Continuous	Ac, NB, P4, P6	Key register addresses and buildings (BAG), Cadastre NL	Vector dataset (point/ polygon) containing more than 10 million buildings and 9,3 million addresses (2020) on a scale starting from 1:2.500
	including:				
	-houses, buildings, berths, beach pavilions, caravans, trailers, etc.				
	-utilization function				
	-construction year				
	-building area				
	Education	2018	A(r), NB, P4, P6	Dienst Uitvoering Onderwijs (DUO) - Ministry of Education, Culture and Science	Coordinates and address data per school / institution. Data can be spatially summarized per indicated exposure zone
	-primary schools				
	-secondary schools				
	-special schools				
	-higher education				
	Key statistics 4-digit postal code (a.o. sex and age of inhabitants, household composition, migration background)	1998–2018	P4	PC4 statistics - Statistics Netherlands (CBS)	Available variables for PC4 and PC6 zones can differ. PC4 contains additional statistics from 2015 onwards
	Other statistics 4-digit postal code (accessibility, childcare, facilities culture, -education, -health care, -sport, housing benefits/stock, income, land use, livability, living environ-ment typology, offices, retail and businesses, post offices, travel time, transactions/house prices)	1990–2015 (range can differ per variable)	P4	Miscellaneous (a.o. ABF Research, SWING Real Estate Monitor, Statistics Netherlands (CBS), Dutch Ministry of the Interior and Kingdom Relations)	
	Key statistics 6-digit postal code (a.o. demographics, income, immigrants, housing stock)	2004, 2010	P6	PC6 statistics—Statistics Netherlands (CBS)	Purchased data
	Key statistical figures	2000–2018	Ac, NB, P4, P6	Vierkantstatistieken Statistics Netherlands (CBS)	The CBS dataset ‘vierkantstatistieken’ contains basic statistics on number of inhabitants, dwellings, residential density and urbanity for all years and additional statistics from 2011 onwards
	per 100 x 100 meter grid cell				
	Number of inhabitants				
	Inhabitants < 15 years				
	Inhabitants 15–25 years				
	Inhabitants 25–45 years				
	Inhabitants 45–65 years				
	Inhabitants > 65 years				
	Total number of men				
	Total number of women				
	Percentage classes:				
	Native Dutch				
	Migr. backgr—western				
	Migr. backgr—nonwestern				
	Number or dwellings				
	Property values				
	Other statistics (households, property age classes, owned/ rented property, single/ multiple family dwellings, social security, energy use number of ca. 30 different destinations within 1/2/ 3 km, distance to nearest destinations (ca. 30))	2015–2018			
	Poverty in % ‘poor’ households	2017	NB, P4	The Netherlands Institute of Social Research (SCP)	Percentage of ‘poor’ households according to SCP definitions per PC4 area and neighbourhood
	Socio-economic status score	1998 2002 2006 2010 2014 2016 2017	P4 (NB 2016)	The Netherlands Institute of Social Research (SCP)	Socio-economic status scores are based on: education, income and position in the labour market)
6. Safety, aesthetics, air temperature	Temperature per km grid	1961-current (daily per year	Ac, NB, P4, P6	Royal Netherlands Meteorological Institute (KNMI)	1 × 1 km grids of interpolated data (Inverse Distance Weighted interpolation, with 2.0 power parameter, block size 20 km and search radius of 110 km) based on 33–35 automatic KNMI observation stations
	-Daily average				
	-Daily minimum				
	-Daily maximum				
	Traffic incidents	Yearly 2003–2017	P6	Bestand geRegistreerde Ongevallen Nederland (BRON)	Provided via ESRI Nl datasets

For each environmental variable and/or geodataset listed in Table 1, a more detailed metadata description is available via http://www.gecco.nl/exposure-data-1. Three examples can be found in Annexes S1–S3 Additional file 1: Annex S1, Additional file 2: Annex S2, Additional file 3: Annex S3.

## Utility and discussion

Interdisciplinary research and collaboration can provide substantial benefits to scientists, practitioners and policy makers and it is predicted that the future of research is increasingly interdisciplinary [33]. GECCO is a solid infrastructure that facilitates such interdisciplinary research. It uses a systematically and integrated method to centralize rigorous and validated scientific information about environmental conditions and exposures. GECCO facilitates the linkage of these data to deep-phenotyped individual-level cohort data enabling identification of spatial or temporal relationships between the exposures and (adverse) health conditions. Besides being an infrastructure, GECCO also supports essentially needed interdisciplinary collaboration as Health Scientists, Epidemiologists (clinical and environmental), Data scientists, Geographers, health cohorts, and GGHDC are involved. Without such collaboration it would be impossible to manage the complexity that arises with integrating data from different disciplines.

### Intended use

Usage of GECCO data is, in principle, free of charge for non-commercial users. A simple GECCO Data Access and Publication Policy has been set up. There are roughly two ways through which the data can be accessed: (1) Centrally, accessible via the GECCO steering group via the website http://www.gecco.nl, or (2) De-centrally, when environmental data linked to individual-level GECCO cohort data is required. As cohorts are enriched with environmental exposures locally (i.e. at the premises where the individual cohort data are stored), usage should be approved by the GECCO steering group, and can be obtained via the respective cohort(s), where additional cohort-specific data sharing regulations need to be complied with. The 23 cohorts that are currently affiliated with GECCO have solid procedures set up for data sharing and use, and must ensure that informed consent procedures allow for that, as specified in the European General Data Protection Regulation (GDPR). The GDPR may provide further challenges with data logistics around analyses. Analyses across cohorts can be done in a number of ways: (1) Pooling cohorts and harmonising variables centrally (if cohorts allow, which is usually not the case); (2) Doing the analyses locally (i.e. without the data leaving the premises of the owners) and meta-analysing results; (3) Accessing data via a so-called trusted third party (TTP); (4) using privacy sensitive data obfuscation [34]; or (5) Federated node analyses. The GECCO consortium has gained experience in handling and combining multiple data sets and cross-cohort analyses have been done successfully within GECCO [6, 35, 36].

### Challenges and options for improvements

The innovation provided by the GECCO database is its extensive coverage (whole population of The Netherlands) and availability of an integrated, large set of personal exposures, ranging from the socio-economic environment to the physical environment. We continuously strive to further improve the database regarding the range of exposures included and the quality of the exposure data. We conduct methodological studies to explore what operationalisations may best reflect real-world exposure e.g. to the food environment [37], and what spatial area to consider [29]. These innovations and the long-term sustainability are guaranteed by ongoing cooperation with partners in the Dutch Global Geo Health Data Center, the Exposome-NL project, the Upstream Team, and the European SURREAL project, among others. We will jointly innovate the methodologies presented here and use exposure data sets in various epidemiological studies.

Envisioned innovations of the data provided by the GECCO database include improvements of the personal exposure calculation and the temporal range of exposures provided. Promising for the improvement of the quality of exposure data is the wider availability of even more detailed maps of environmental factors as well as more advanced exposure assessment methods. The ongoing increase in the volume and spatio-temporal detail of environmental sensor data will lead to more detailed maps of environmental factors in The Netherlands, but also worldwide. Earth observation data collected from space will contribute to hyper resolution mapping in space and time of environmental variables such as air temperature [38], air pollution [39], and green space [40]. Near sensing data collected close to the land surface, provide a wealth of information thus far not used in the GECCO data base. Future improvements could include the assessment of the attractiveness of the living environment (e.g. green space) from street view imagery [41, 42], and the use of dense networks of low cost (mobile) sensors for air pollution mapping in space and time. A more novel and yet to be harnessed data source for environmental epidemiology is the data continuously gathered by modern cars that are both connected to internet and equipped with sensors to map the environment—for safety interventions such as emergency stops, and functions such as autonomous driving. By using advanced spatio-temporal machine learning algorithms, the remote and near sensing innovations will lead to environmental attribute data at a higher spatial resolution, as well as data representing temporal changes, for instance diurnal or seasonal patterns of air temperature [38] or air pollution [43]. This improved resolution and coverage of environmental data will contribute to the development of more sophisticated environmental exposure assessment methods. One innovation is to replace spatial buffers to represent activity spaces of persons by methods that give a more detailed representation of the activity of persons in space and time, by activity-based or agent-based modelling [44]. Another requirement to improve exposure assessment is to make exposure assessment specific for the actual space–time activity patterns of persons, for instance using GPS wearables. Alternatively, exposure assessment parameters between different groups of persons can be based on their typical daily movement pattern, for instance homemakers, students, or commuters (e.g., [45]).

Furthermore, qualitative individual-level data could be integrated with the objectively measured GIS data. Geographic information systems are considered to be a tool for the storage and analysis of quantitative data, but there are examples of their use in qualitative or mixed-method research [46, 47]. This would add contextual information on factors that potentially co-determine health outcomes.

An additional path of innovation is to expand the data set with more temporal data. Personal environmental exposure can be considered as integration of exposures over an interval of time. The interval of time that is relevant may depend on the health outcome considered. For instance, the influence of air pollution or food outlet exposures on many cardio vascular disease outcomes is considered to be a long-term process, and one would require exposure values calculated over time spans of several years or even from conception onwards. Other health outcomes are more instantaneous, for instance the occurrence of hay fever due to pollen in the air in which case personal exposures are required integrated over a few hours to days. To deal with these situations, temporal databases of environmental factors are required, something which has only partly been addressed in our current database.

While GECCO is part of international projects and networks such as the Initiative on Spatial Lifecourse Epidemiology (ISLE) [48, 49], a relevant step for the (near) future would be a better alignment of measures and methods with similar infrastructures elsewhere in the world. For instance, the Canadian Urban Environmental Health Research Consortium (CANUE) has similarities as it was established to facilitate the linkage of extensive geospatial exposure data to existing Canadian cohorts and administrative health data holdings [50]. The potential Exposome studies across countries or even continents require standardisation and harmonisation, and stresses the need for continuance or solid embedding of such infrastructures in sustainable programs that are less dependent on temporary funding.

GECCO has not been set up to address a specific research question but is rather an infrastructure to address a myriad of questions, also beyond the types of examples that are provided in this manuscript. The relevance of—and the forms of approaches to address—such questions are likely to evolve over time. The relatively novel area of environmental epidemiology and exposome research is developing rapidly and will need to cope with changes of exposures, whether they are gradual [51] or very swift e.g. due to covid-19, where actual exposures changed [52], but also spatial patterns of people within contexts of exposures.

## Conclusions

The systematic approach of the GECCO infrastructure to centralise environmental data and develop personal exposure variables at high resolution across various domains has resulted in a large, accessible and utilisable source for exposome research. Particularly harnessing the increasing availability of—and accessibility to—remote and near sensing data as well as alignment with other similar infrastructures globally are identified as key next steps for further improvement.

## Supplementary information

**Additional file 1.** Annex S1.

**Additional file 2.** Annex S2.

**Additional file 3.** Annex S3.

## Data Availability

All environmental exposure data described in this study are available through GECCO but restrictions or conditions may apply to the availability of these data, as some are under license, and so are not publicly available. Data are available from the authors upon reasonable request and with permission of the GECCO consortium. A data request form can be downloaded via https://www.GECCO.nl/exposure-data-1/. Most datasets described are derived from data that are available in the repositories as listed in Table 1 (Geodata source).

## Abbreviations

BRON: Bestand geRegistreerde Ongevallen Nederland
CBS: Statistics Netherlands
DANS: Data Archiving and Networked Services
dB: Decibel
DSA: Databestand SportAanbod
DUO: Dienst Uitvoering Onderwijs
ESCAPE: European Study of Cohorts for Air Pollution Effects
FGDB: File Geodatabase format
GDPR: General Data Protection Regulation
GECCO: Geoscience and hEalth Cohort COnsortium
GeoDMS: Geo Data and Model Software
GGHDC: Global Geo Health Data Center
GIS: Geographic information system
GPS: Global Positioning System
IRAS: Institute of Risk Assessment Sciences
MAUP: Modifiable Areal Unit Problem
NCD: Non-communicable diseases
NDW: National Data Warehouse
NGR: Nationaal Geo Register
PBL: Dutch environmental assessment agency
PC4: 4-digit postal code
PC6: 6-digit postal code
PDOK: Publieke Dienstverlening Op de Kaart
RIVM: National Institute for Health and Environment
SCP: Netherlands Institute for Social Research
TTP: Trusted Third Party
