# Supplementary material for: Deep phenotyping meets big data: the Geoscience and hEalth Cohort COnsortium (GECCO) data to enable exposome studies in The Netherlands
Source: Int J Health Geogr. 2020 Nov 13;19:49. doi: 10.1186/s12942-020-00235-z (PMC7662022; doi:10.1186/s12942-020-00235-z)
Supplement: Supplementary file 1 — Additional file 1. Annex S1. [file 12942_2020_235_MOESM1_ESM.docx]

**ANNEX S1**

**Meta data: Bicycle roads surface 2019**

**Spatial scale / resolution:** PC6 Address / point coordinates

**Spatial coverage:** Netherlands

**Temporal range:** 2019

**Data format input data:** ESRI File Geodatabase (FileGDB) and ESRI Shape files (*.shp)

**Data format output data:** Polygons/Lines / ESRI File Geodatabase (FileGDB)

**Data source input data:** BGT 2019, TOP10 2019, kaarten.routedatabank.nl
(Landelijk Fietsplatform)

**Data storage outputdata:** ..\Source_data\BGT_fietspaden\*

..\TOP10_gemengd_verkeer_buf1m_splitbuurt2016_erase_fkpnbuf10.shp

..\TOP10_fietswegen_splitbuurt2016_erase_fkpnbuf10.shp

..\BGT_fietspaden_splitbuurt2016_erase_fkpnbuf10.shp

..\Fietsknooppuntnetwerken_splitbuurt2016.shp

..\Fietspadoppervlak_BGT_TOP10fw_GV_fkpn_Buurten2016.shp

**Data description:**

This dataset combines bicycle paths from the following input sources:

1. Separate bicycle lanes (‘fietspaden’) from polygon feature class ‘wegdeel’ in the Basisregistratie Grootschalige Topografie ( BGT ) 2019. These bicycle lanes are physically separated from roads. e.g. by a strip of unpaved land, a ditch, a fence or another separation different from a painted marking on the street or different road material see <http://imgeo.geostandaarden.nl/def/imgeo-object/wegdeel/fietspad> for details
2. TOP10 2019 roads. This concerns the line feature class ‘Wegdeel Hartlijn’, attribute field HOOFDVERKEERSGEBRUIK = ‘fietsers, bromfietsers’ OR ‘gemengd verkeer’.
3. Fietsknooppuntnetwerken from ‘kaarten.routedatabank.nl’ (Landelijk Fietsplatform). This is a line dataset based on (a generalized version of) the heartlines from the TOP10 2019 roads dataset.

Because the input data is a combination of line and polygon data, we have chosen to convert all data to a uniform polygon format in which the different bicycle paths or routes are assigned different widths. We have based the polygon width of the bicycle paths on their actual width or, if unknown, on the basis of their supposed cyclability and/or importance in the bicycling network:

BGT bicycle paths: ca. 2 – 4 meters (original BGT values)

TOP10 fietswegen (specific bicycle roads or lanes): 3 meters (1.5 meter buffers)

TOP10 gemengd verkeer (mixed traffic, including bicycles): 1 meter (0.5 meter buffers)

Fietsknooppuntnetwerken (bicycle junction networks) : 4 meters (2 meter buffers)

An important characteristic of all bicycle path maps except the *Fietsknooppuntnetwerken* is that the paths are separated with a distance of 10 meters from the *Fietsknooppuntnetwerken*, see the map example below in which the orange and grey lines are respectively the TOP10 *fietswegen* and the TOP10 *gemengd verkeer* and the bold green line is the *Fietsknooppuntnetwerken*. The reason for these gaps in the bicycle paths is the construction method for these maps in which duplicate parts of overlapping bicycle paths had to be removed with an additional buffer distance, due to generalization differences between the different source maps.


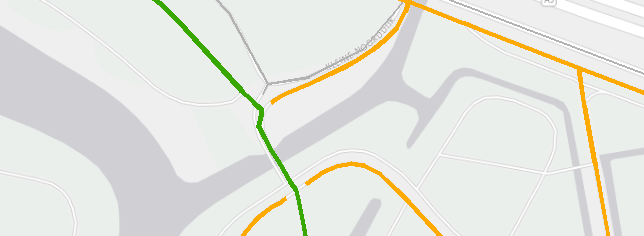


After the production of the 4 polygon bicycle path maps, we have combined and spatially summarized them in a spatial join operation to a neighborhood surface density map for the neighborhood borders of the year 2016. We have produced different attribute fields containing the total bicycle area per neighborhood and the bicycle area density as a percentage per neighborhood. We have produced these fields for all bicycle paths combined and for a combination without the mixed traffic roads. See the data processing section and map examples and the description of variables for a better understanding of the different datasets.

**Data processing:**

Our objective is to create a line dataset of different classes of possible bicycle routes on shared roads or separate bicycle lanes that are part, or not part, of a national cycling network. To select separate, dedicated bicycling lanes I use the polygon dataset from BGT in which cycling lanes (‘fietspaden’) are a separate feature class. Other, cycling lanes and mixed traffic roads for cycling I select from the TOP10 dataset, while I use the Fietsknooppuntnetwerken data from kaarten.routedatabank.nl, to indicate if cycling lanes are part of the national cycling network.

To differentiate between different types of bicycle paths I used different buffer widths. This implies I use the width of the bicycle path (which is in most cases not the real width, at most a relative width) to indicate its relevance in the bicycle path network.

BGT bicycle paths: ca. 2 – 4 meters (real) mapped width according to BGT

TOP10 fietswegen: 3 meters (1.5 meter buffers on both sides of lines)

TOP10 gemengd verkeer: 1 meter (0.5 meter buffer on both sides of lines)

Fietsknooppuntnetwerken: 4 meters (2 meter buffer on both sides of lines)

The rationale behind these indicative widths is that I consider the fietsknooppuntnetwerken the most important thoroughfares / main roads for bicycle travel (even though these routes do not always have physically separated bicycle paths and can be separated only by painted road lining) , followed by the BGT bicycle paths (physically separated from other road sections by distance or delimitation other than painted lines and/or road color), TOP10 fietswegen (exact definition unknown, I assume some kind of delimitation including road colors and/or painted lines) and TOP10 gemengd verkeer. On the gemengd verkeer road class bicycles are allowed, but cycling on these roads is less attractive because of higher traffic risks and pollution than on separated bicycle paths. Therefore I gave this class a smaller road width of 1 meter only.

Selections

TOP10 line feature layer WEGDEEL_HARTLIJN

=> all records from attribute class Hoofdverkeersgebruik = 'fietsers, bromfietsers'

=> export to a separate dataset named ‘Fietswegen_TOP10_hartlijnen_2019.shp’

TOP10 line feature layer WEGDEEL_HARTLIJN

=> all records with attribute class ‘gemengd verkeer’

=> export to a separate dataset named ‘Gemengdverkeer_TOP10_hartlijnen_2019.shp’.

BGT 2019 polygon feature layer WEGDEEL

=> all records with attribute class ‘Fietspad’

=> export to a separate dataset named ‘Fietspaden_BGT2019.shp’.

Line – polygon conversion

The selections of the TOP10 line layers and Landelijk Fietsplatform are converted to polygons by buffering them.

- Buffer Fietswegen_TOP10_hartlijnen_2019 with 1.5 meter (to simulate 3 meter wide bicycle paths)
- Buffer Gemengdverkeer_TOP10_hartlijnen_2019 with 0.5 meter (to simulate 1 meter wide bicycle lanes)
- Buffer SQL_fietsknooppuntnetwerken with 2 meter (to simulate 4 meter wide bicycle paths)

Union - neighborhood split

To split polygons at neighborhood borders and places where bicycle paths from BGT and TOP10 overlap (so redundant parts can be removed later on) unions between different layers were carried out.

- Union between [Fietswegen_TOP10_hartlijnen_2019], [Fietspaden_BGT2019] and [Buurt2016]
- Union between [Gemengdverkeer_TOP10_hartlijnen_2019] and [Buurt2016]

- Export splitted BGT polygons to BGT_fietspaden_splitbuurt2016.shp
- Export splitted TOP10 polygons to TOP10_fietswegen_splitbuurt2016.shp
- Export splitted TOP10 polygons to TOP10_gemengd_verkeer_splitbuurt2016.shp

Erase duplicates

To remove duplicate bike path polygons from BGT that are already represented by bike path polygons from the Fietswegen TOP10, I have created a buffer layer from Fietswegen_TOP10_hartlijnen_2019 with a buffer distance of 5 meters on all sides (Fietswegen_TOP10_hartlijnen_2019_buf10m.shp) and used this layer to erase most parts of the BGT bike path polygons that coincided with the fietswegen TOP10 layer.

The resulting layer is named ‘BGT_fietspaden_splitbuurt2016_selection1_erase_fietswegen-TOP10_buf10m.shp’.

This layer I dissolved on the fields ‘Id’ and ‘Buurtnaam’, after which I removed all polygons < 50 m2 and joined the attributes of the original layer, resulting in the layer ‘BGT_fietspaden_splitbuurt2016_selection1_erase_fietswegenTOP10_buf10m_dissolve_GT50m2_-joinattributes.shp’.

Resulting layers

The result of these operations are 4 different polygon layers splitted by the neighborhood map of 2016 (‘buurt_2016.shp’):

- BGT fietspaden with variable widths, approximately between 2 meters and 4 meters wide, the first being one direction paths and the latter two direction paths.
- TOP10 ‘fietswegen’, buffered to 3 meters wide.
- TOP10 ‘gemengde wegen’, buffered to 1 meter wide.
- Fietsnetwerken Landelijk Fietsplatform, buffered to 4 meters wide.

Layer corrections and updates

The layer ‘Fietsnetwerken’ contains routes outside the national borders, which are selected and removed.

The ‘fietsnetwerken’ layer overlaps for a large part with the TOP10 ‘fietswegen’, the TOP10 layer ‘gemengd verkeer’ and the BGT fietspaden layer. Unfortunately, there is no good way of removing or replacing overlapping sections in a neat way because the ‘fietsnetwerken’ layer is based on a generalized version of the TOP10 heartlines, which means that the spatial overlap between roads from the TOP10 and from the fietsnetwerken layer is only partial. For example highly curved roads in the ‘fietsnetwerken’ layer follow a different more simplified heartline than the TOP10 heartlines, see the left map in the figure here below. Because I want to give preference to the ‘fietsnetwerken’ layer, I have created a buffered version of this layer with a 10 meter buffer and use this buffered layer to erase polygons from the TOP10 ‘fietswegen’, the TOP10 layer ‘gemengd verkeer’ and the BGT fietspaden layer. This way, there will be no more overlap between the TOP10 / BGT layers and the ‘fietsnetwerken’ layer. But as a consequence a (smaller) error is introduced because the buffer removes as well small connecting sections between TOP10 /BGT bicycle paths and paths from the ‘Fietsnetwerken’ layer, see the missing road section in the blue circle in the right map of the figure below.


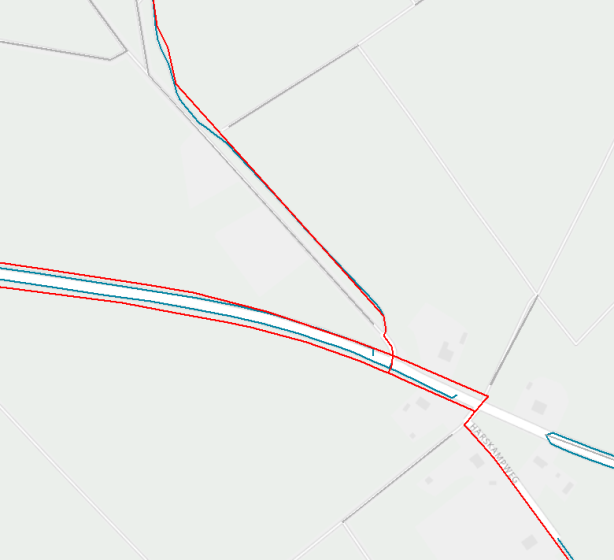

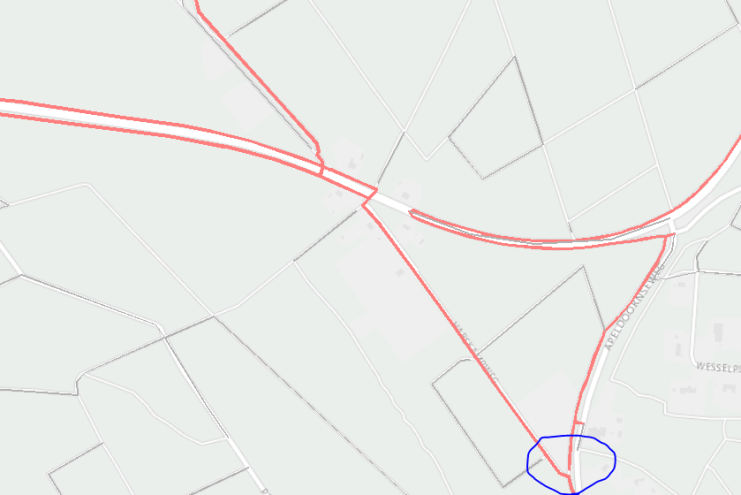

*In blue fietswegen TOP10 and in red fietsknooppuntnetwerken*

Spatial joins between bicycle path layers and neighborhood layer

Finally, the polygon areas for each bicycle path class were summed in four consecutive spatial joins between buurt2016 and each of the 4 splitted bike path layers, see example below for the last join. In each spatial join the match option (‘CONTAINS’, do not choose intersect, this will also select features of neighbouring polygons!) and the merge rule ‘SUM’ is used for the added join features. In this way the following fields have been created in the resulting join layer named ‘Fietspadoppervlak_BGT_TOP10fw_GV_fkpn_Buurten2016.shp’:

m2_BGT: Area BGT bike paths in m^2^ per neighborhood

m2_T10fw: Area TOP10 Fietswegen (bicycle roads) in m^2^ per neighborhood

m2_T10GV: Area TOP10 Gemengd verkeer (mixed roads) in m^2^ per neighborhood

m2_fkpn: Area bike paths Fietsnetwerken Landelijk Fietsplatform in m^2^ per neighborhood


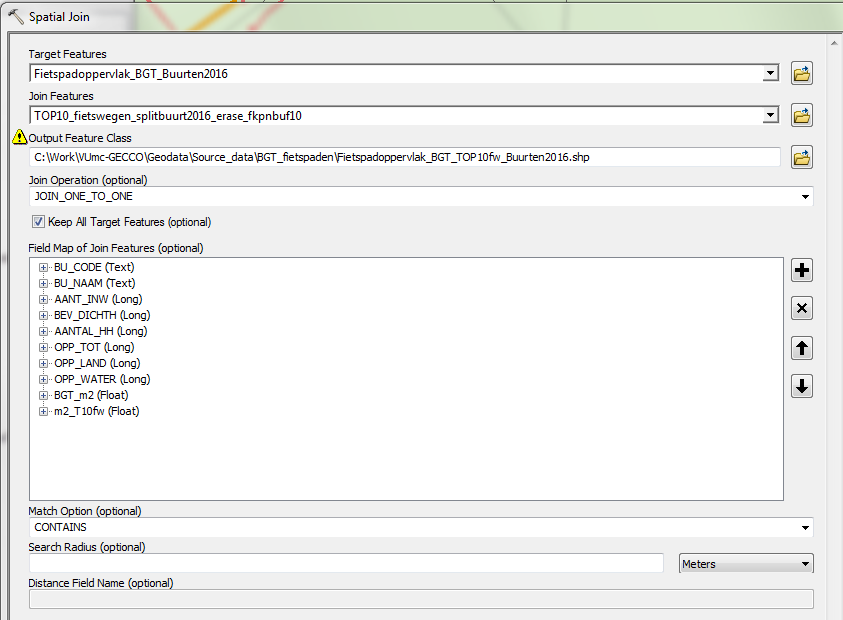

*Example input settings for spatial join operation*

Calculate total bicycle path areas and densities

The total bicycle path area per neighborhood INcluding mixed roads is calculated in a new field named ‘m2_tot’:

[m2_tot] = [BGT_m2] + [m2_T10fw] + [m2_T10GV] + [m2_fkpn]

The total bicycle path area per neighborhood EXcluding mixed roads is calculated in a new field named ‘m2_tot’:

[m2_tot_BP] = [BGT_m2] + [m2_T10fw] + [m2_fkpn]

The bike path density per neighborhood INcluding mixed roads is calculated in a new field named ‘BP_dens’:

100* ([m2_tot] / ([OPP_LAND] *10000))

The bike path density per neighborhood EXcluding mixed roads is calculated in a new field named ‘BP_dens_z’:

100* ([m2_tot_BP] / ([OPP_LAND] *10000))

**Map examples**

..\Geodata\Source_data\ BGT_fietspaden \**.mxd


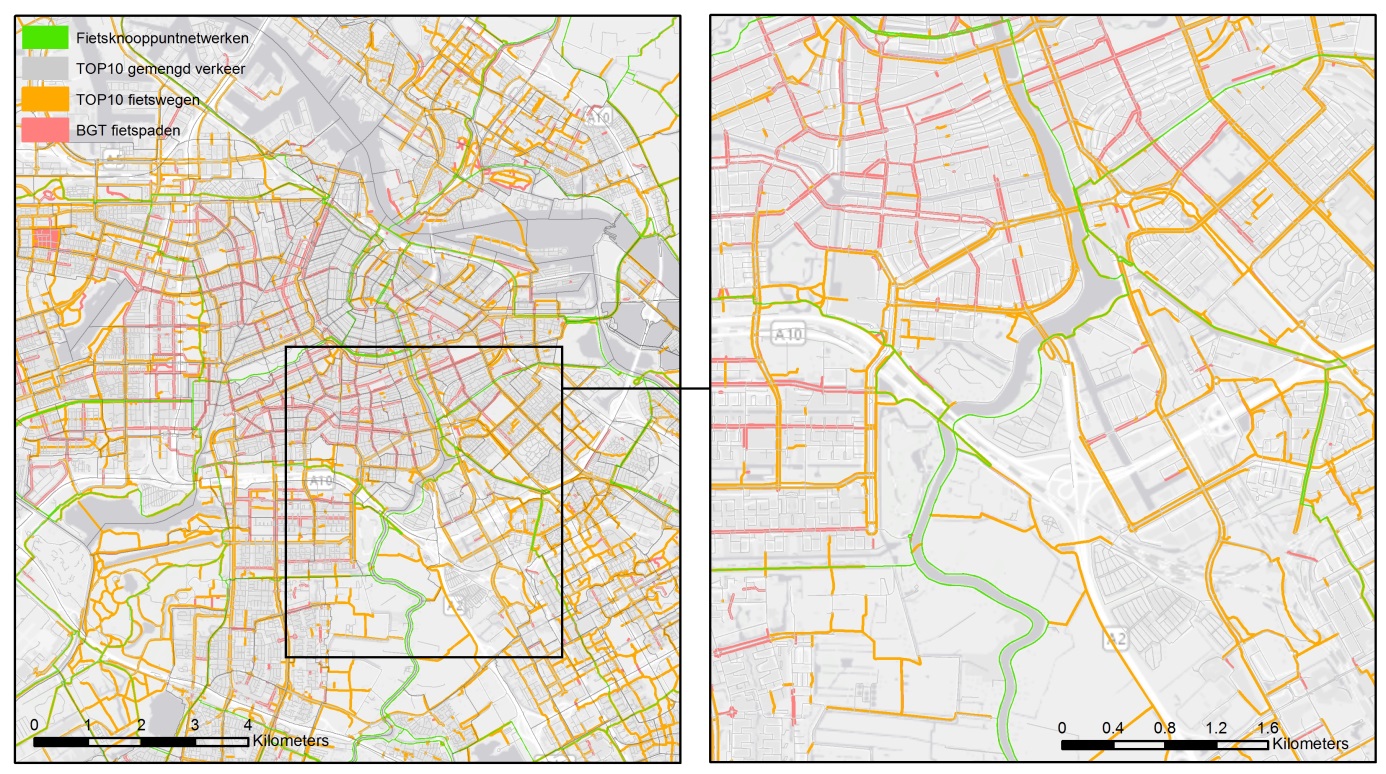


*Bicycle path polygon maps TOP10, BGT and Fietsknooppuntnetwerken*


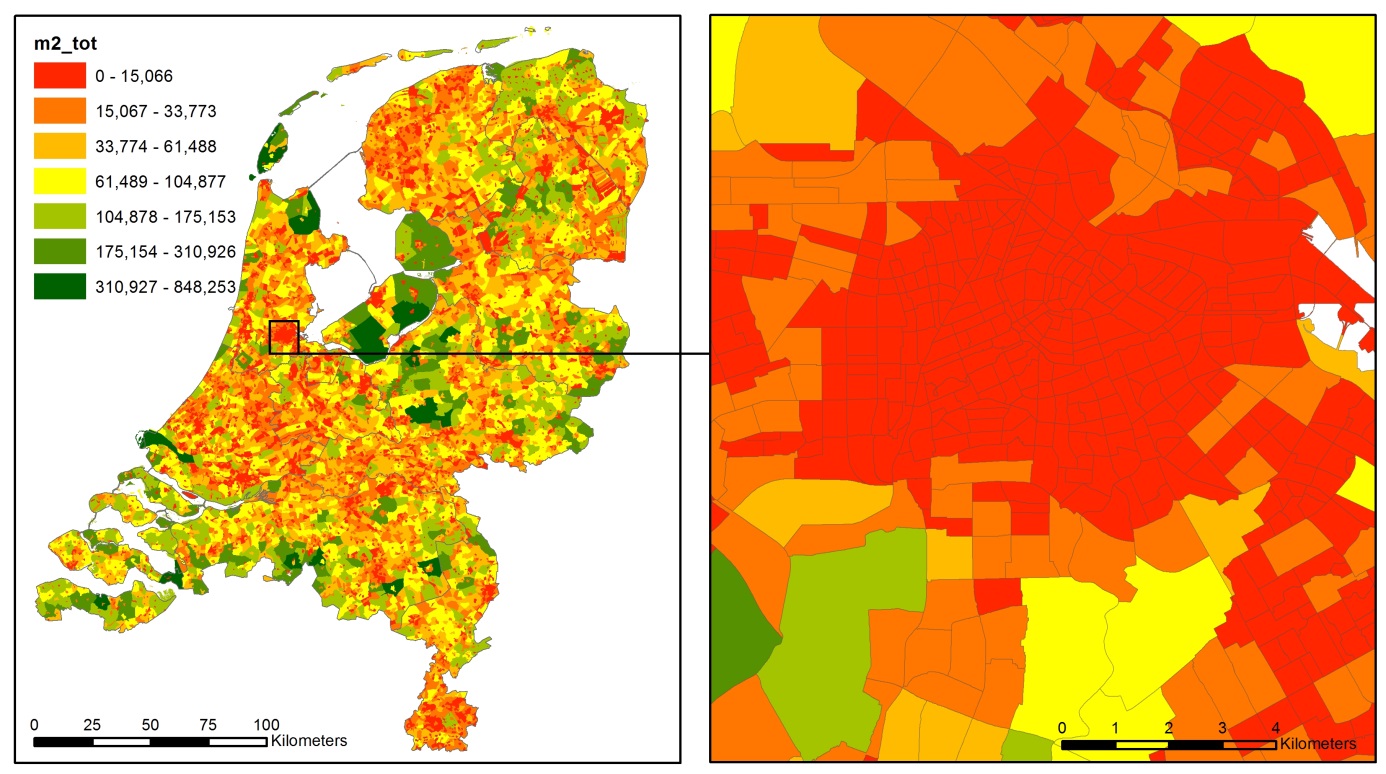


*Bicycle path area in m^2^ per neighborhood (****in****cluding mixed roads). Classification: Natural breaks (Jenks)*


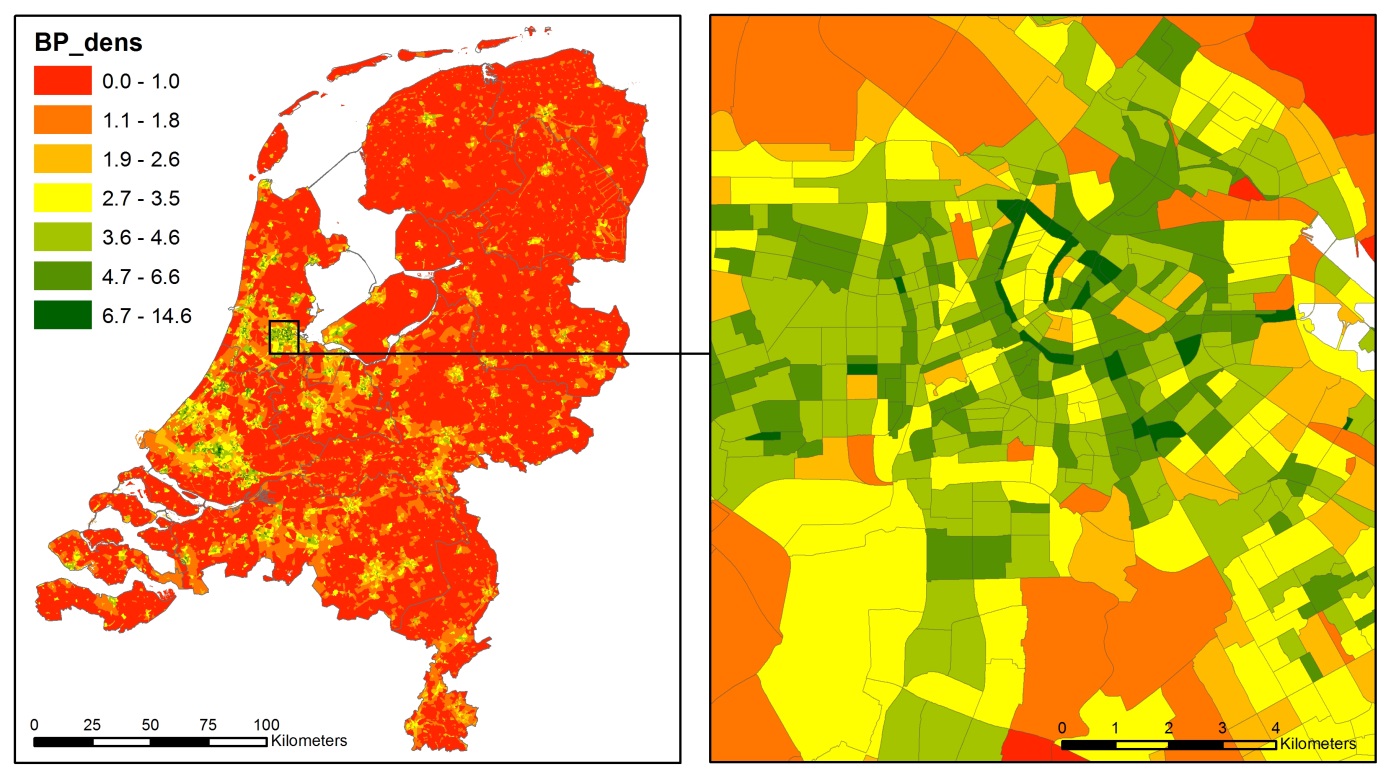


*Bicycle path density* ***IN****cluding mixed roads as % per neighborhood. Classification: Natural breaks (Jenks)*

*
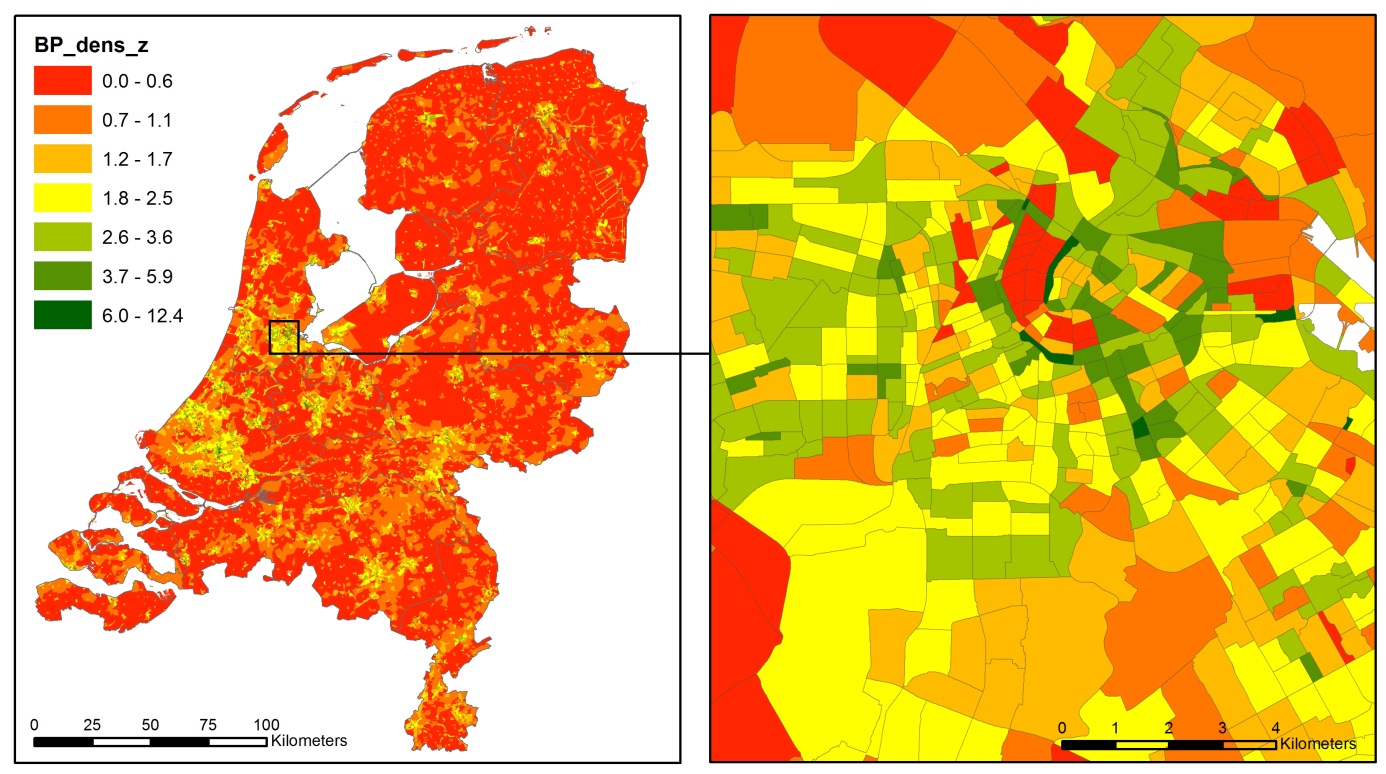
*

*Bicycle path density* ***EX****cluding mixed roads as % per neighborhood. Classification: Natural breaks (Jenks)*

**Variables**

Table 1 on the next page provides an overview of variables that are available in this dataset.

**Table 1: Overview of attribute data in Fietspadoppervlak_BGT_TOP10fw_GV_fkpn_Buurten2016.shp**

| **Variable name** | **Description** | **Original dataset** |
| --- | --- | --- |
| BU_CODE | Neighborhood code | buurt_2016 |
| BU_NAAM | Neighborhood name | buurt_2016 |
| AANT_INW | Number of inhabitants per neighborhood | Buurt2016 |
| BEV_DICHTH | Population density | Buurt2016 |
| AANTAL_HH | Number of households per neighborhood | Buurt2016 |
| OPP_TOT | Total neighborhood area water and land in hectare | Buurt2016 |
| OPP_LAND | Total neighborhood area land in hectare | Buurt2016 |
| OPP_WATER | Total neighborhood area water in hectare | Buurt2016 |
| BGT_m2 | Area bicycle paths BGT in m2 | Fietspadoppervlak per buurt 2016 |
| m2_T10fw | Area bicycle paths TOP10 roads in m2 | Fietspadoppervlak per buurt 2016 |
| m2_T10GV | Area mixed roads TOP10 in m2, including cyclists | Fietspadoppervlak per buurt 2016 |
| m2_fkpn | Area bicycle paths fietsknooppuntnetwerken in m2 | Fietspadoppervlak per buurt 2016 |
| m2_tot | Total area bicycle paths in m2 | Fietspadoppervlak per buurt 2016 |
| m2_tot_BP | Total area bicycle paths without mixed roads in m2 | Fietspadoppervlak per buurt 2016 |
| BP_dens | Area density bicycle paths per neighborhood in % | Fietspadoppervlak per buurt 2016 |
| BP_dens_z | Area density bicycle paths, excluding mixed roads, per neighborhood in % | Fietspadoppervlak per buurt 2016 |

**Data providers**

BGT 2019 - Basisregistratie Grootschalige Topografie: Kadaster

TOP10 NL 2019 - Basisregistratie Topografie - BRT: Kadaster

Fietsknooppuntnetwerken: Landelijk fietsplatform

**Data quality**

See description data processing

**Contact information**

GECCO - Geoscience and health cohort consortium

Department of Epidemiology and Biostatistics

Location VUmc, De Boelelaan 1089a, 1081 HV Amsterdam

E: [j.lakerveld@amsterdamumc.nl](mailto:j.lakerveld@amsterdamumc.nl), [a.wagtendonk@amsterdamumc.nl](mailto:a.wagtendonk@vumc.nl)

T: 0031 (0)6 24858837

**Terms and conditions**

None, all public data. Please refer to GECCO when using one or more of these datasets

**Suggested data referencing**

GECCO reference

**List of references**

n.a.
