# Supplementary material for: Deep phenotyping meets big data: the Geoscience and hEalth Cohort COnsortium (GECCO) data to enable exposome studies in The Netherlands
Source: Int J Health Geogr. 2020 Nov 13;19:49. doi: 10.1186/s12942-020-00235-z (PMC7662022; doi:10.1186/s12942-020-00235-z)
Supplement: Supplementary file 2 — Additional file 2. Annex S2. [file 12942_2020_235_MOESM2_ESM.docx]

**ANNEX S2**

**Meta data: Parking spaces per neighbourhood 2019**

**Spatial scale / resolution:** Neighborhoods

**Spatial coverage:** Netherlands

**Temporal range:** 2019

**Data format input data:** Points / ESRI File Geodatabase (FileGDB)

**Data format output data:** Polygons ESRI Shape files

**Data source input data:** ..\Source_data\Traffic\Parking\Park_data2.gdb\ Total_parkspaces_2019 (point dataset)
..\Source_data\Traffic\Parking\Parkeerlocaties-met-specificaties-RDW-opendata-versie-nov2019_GEO-Parkeergarages_capacity_per_entrance_RD_2.shp

**Data storage outputdata:** ..\Source_data\Traffic\Parking\

**Data description:**

The neighborhood maps of parking locations and its derived variables are based on a combination of datasets:

1. Parking areas as polygons in the ‘Basisregistratie Grootschalige Topografie’ (BGT) 2019
2. Parking areas as polygons in TOP10 (Basisregistratie Topografie - BRT) 2015 or 2019
3. Point locations of entrances or exits of parking areas / garages by RDW with parking capacity
4. Specific criteria-based polygons from the BAG dataset (2015) that are interpreted as private parking areas (area between 15 and 40 m2 and < 25 meters distance of existing dwellings)

These datasets are combined in several polygon and point datasets in which parking areas in m2 have been translated to number of parking places. See for more details on the input data, the metadata sheet ‘Metadatasheet - Parking spaces 2019.docx’.

In total we find a number of circa 11.800.000 parking places, of which approximately 8.820.000 are shared parking places on the street, about 403.000 spaces are located in public or semi-public parking garages and approximately 2.577.000 private places can be linked to inhabited dwellings. The number of spaces in public or semi-public parking garages is certainly an underestimated number because the list with parking garages from RDW is far from complete, many commercial garages, e.g. the ones from Parkbee, are missing as well as the many company based –for employees only- garages. Furthermore, the number of 2.577.00 private parking places is uncertain as this number is based on a specific selection of buildings in BAG, based on the area of the building, it’s distance to existing dwellings and the ‘verblijfsobject’ type of the building. The criteria can both include too much and too little buildings that are or can be used as a private car parking.

The point datasets have been input for the production of a series of neighborhood maps in which parking data has been combined with other already available neighborhood data, such as neighborhood area and number of households or number of registered cars, from the CBS buurtkaarten. These concern:

- the total number of parking spaces per neighborhood (see figure 1)
- the number of parking spaces for each household in a neighborhood (see figure 2)
- the number of parking places per hectare per neighborhood (see figure 3)
- total number of registered cars (private & company) by RDW per neighborhood (see figure 4)
- the ratio between cars and parking places per neighborhood (see figure 5)


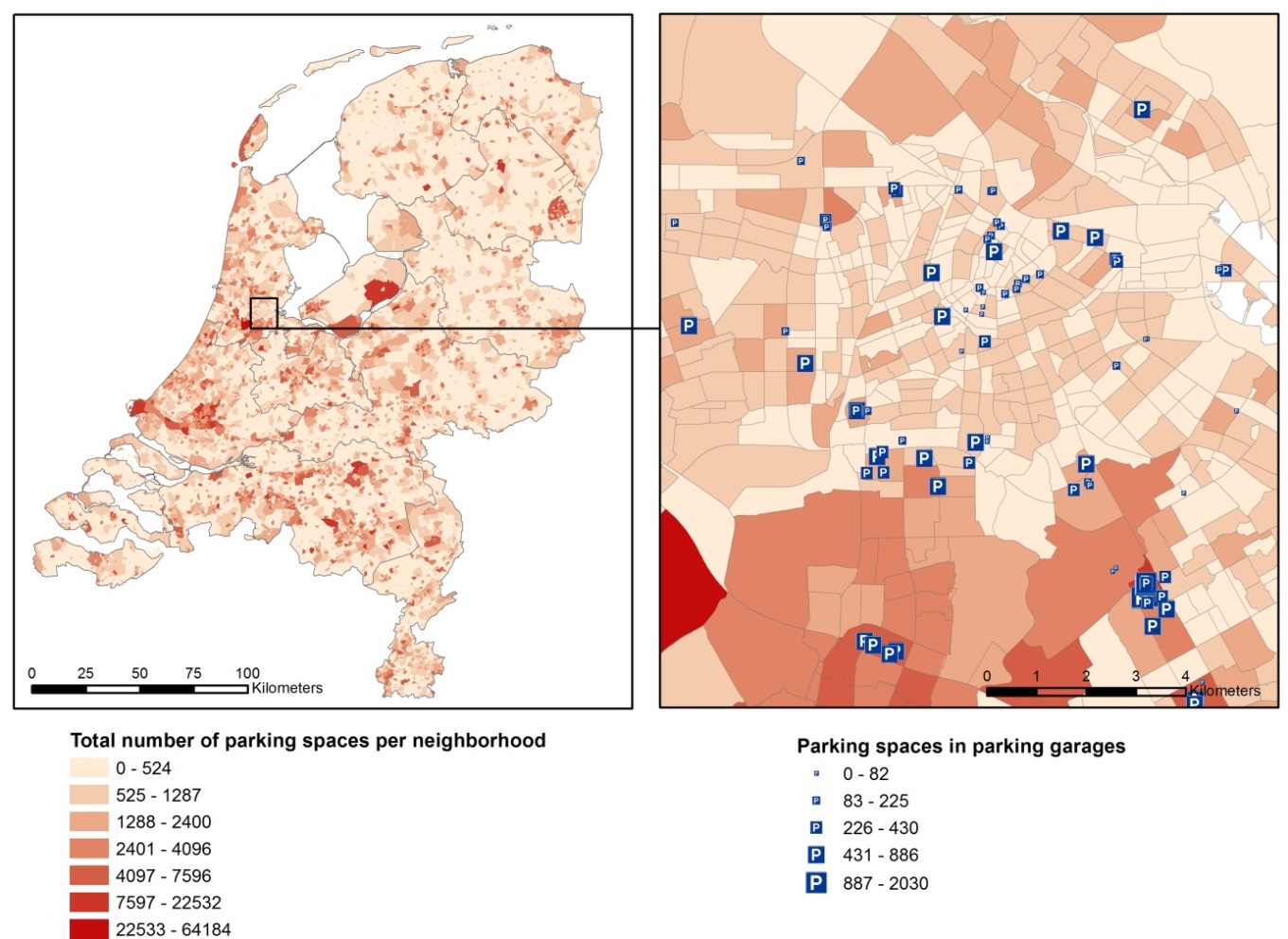


*Figure 1: the total number of parking spaces per neighborhood*


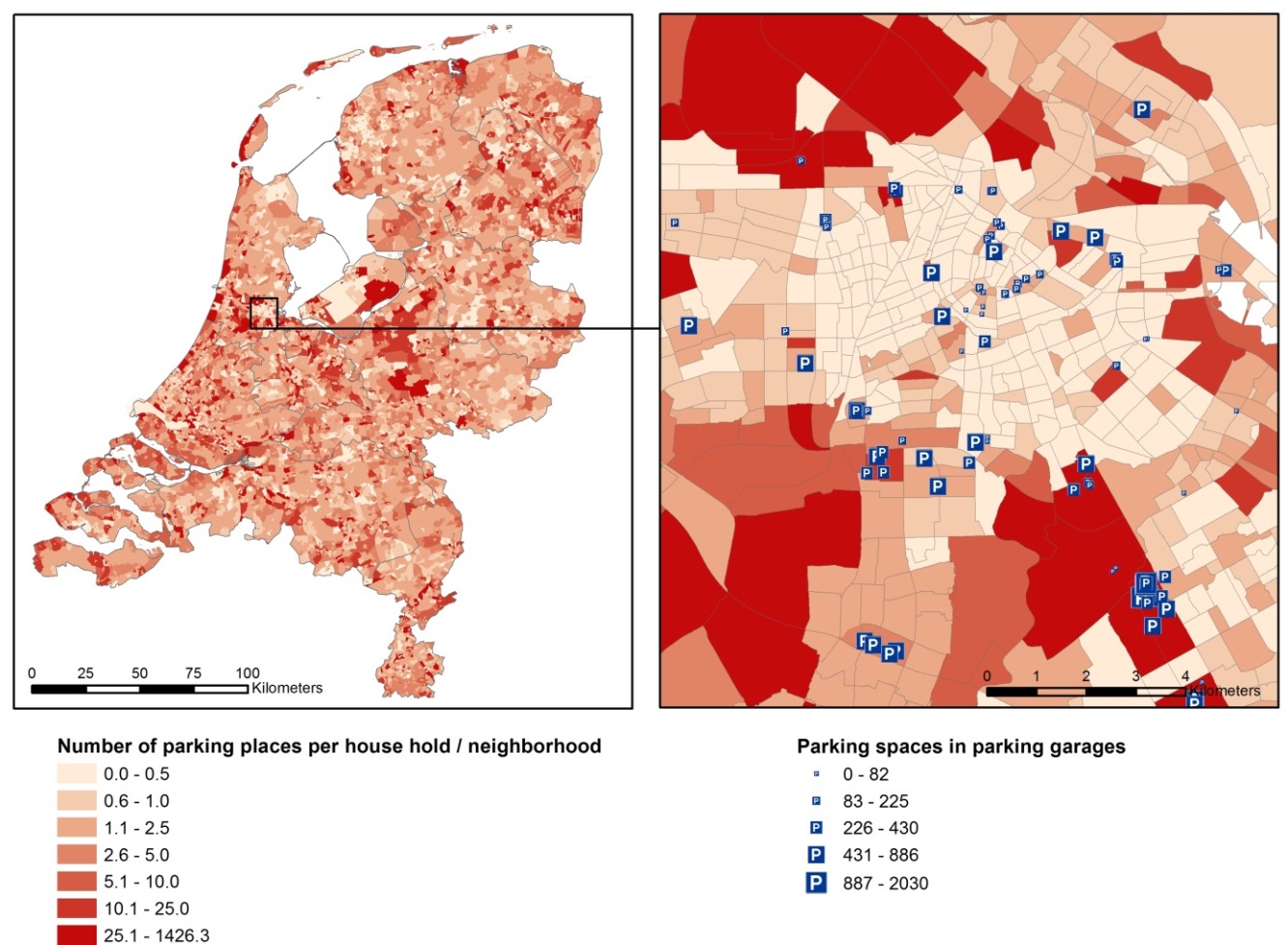


*Figure 2: the number of parking spaces for each household in a neighborhood*

**
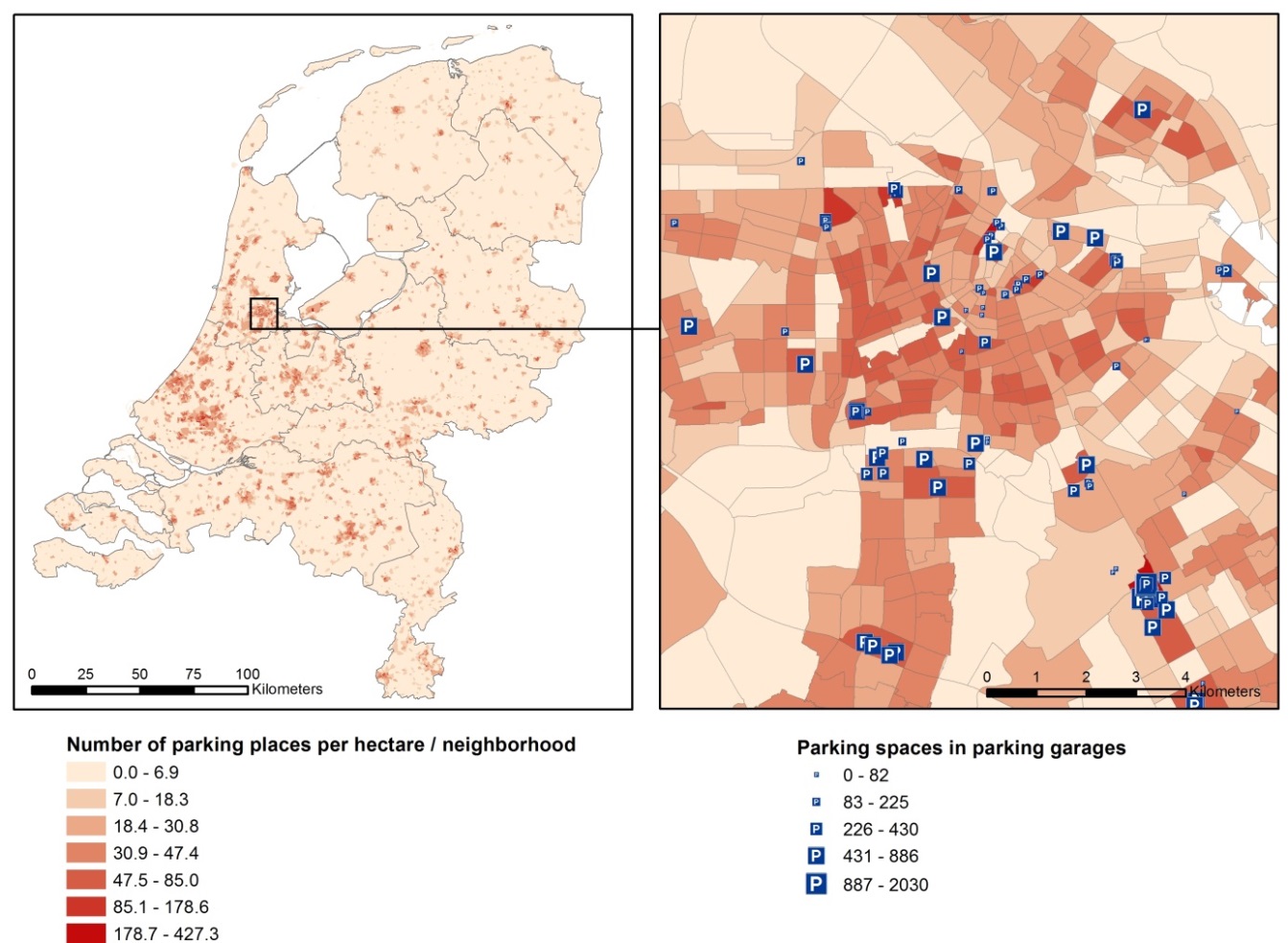
**

*Figure 3: the number of parking places per hectare per neighborhood*

Although figures 1 and 2 with respectively the number of parking spaces per household and the number of parking spaces per hectare are useful, they give little information about the relative availability of parking places (which also depends on parking pressure and other factors such as parking costs which are not considered here). For example, the number of parking places in the neighborhood ‘Amsterdamse bos Noord’ (see map in metadata sheet ‘Metadatasheet - Parking spaces 2019.docx’) is fairly large thanks to some large parking areas at the eastern side of this neighborhood. Nevertheless, because this neighborhood is very big and mainly forest the number of parking spaces per hectare (Figure 3) for this neighborhood is still very low. At the same time, the number of parking spaces per household is very high because there as so few households in this neighborhood. However, because the focus of this study is on the impact of the living environment on individual inhabitants or households, the number of parking spaces per household in a neighborhood seems still valuable information.

Because the CBS buurtkaart Nederland also contains attribute data on car possession, which is relevant data for drivability I added all relevant fields to the neighborhood map which I considered potentially useful. In figure 4 I have added a map showing the number of registered motor vehicles per neighborhood (sum of private and company vehicles) and figure 5 shows the ratio between the number of motor vehicles and parking spaces per neighborhood. Thus areas with more motor vehicles than parking spaces have a ratio > 1 (orange to red colors) and areas with less motor vehicles than parking spaces a ratio < 1 (green to yellow colors). The ratio shows for example that several neighborhoods in the central parts of Amsterdam have ratio’s > 1, thus more cars than available spaces. I expect that in reality the ratio’s will be less high, because my park space map certainly misses a percentage of parking spaces (e.g. private parking spaces in parking garages of companies), but I expect that the relative differences between neighborhoods have a significate meaning. Outside the city, orange and red colors can also be seen in the country side. These colors can be explained by much larger numbers of informal parking spaces, e.g. on unpaved roadsides.

**
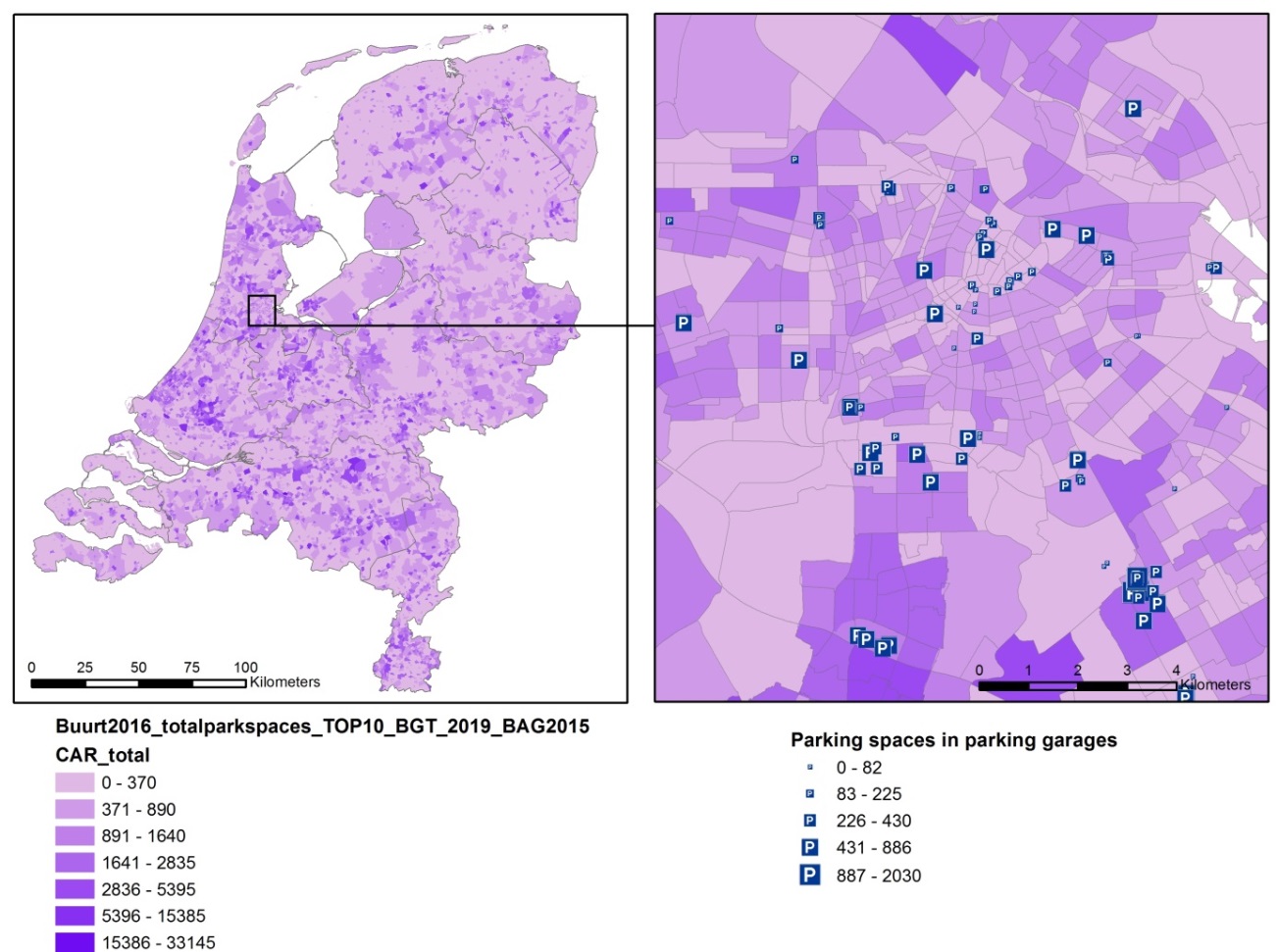
** *Figure 4: the total number of registered cars (private and company cars) by RDW per neighborhood
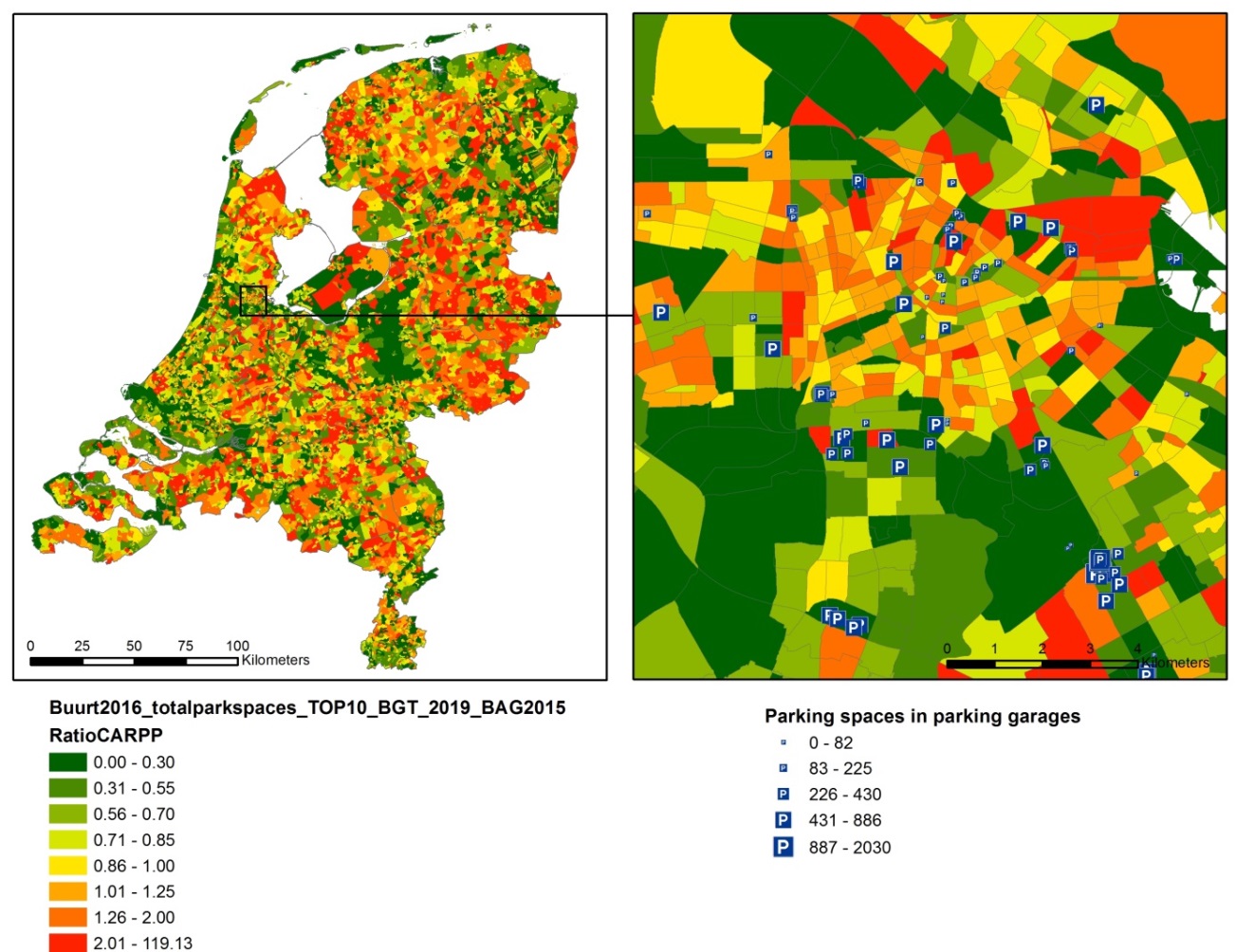

Figure 5: the ratio between cars and parking places per neighborhood*

**Data processing:**

To aggregate the number of parking places per neighborhood I carried out two spatial join operations

**1.** between the neighborhood layer ‘buurt_2016’ (the target layer) and as the ‘join features’ layer the point layer with the BGT-TOP10-BAG parking locations (‘Total_parkspaces_2019’), resulting in layer ‘Buurt2016_totalparkspaces_TOP10_BGT_2019.shp’.

**2.** between the joined neighborhood layer ‘Buurt2016_totalparkspaces_TOP10_BGT_2019.shp’ (the target layer) and as the ‘join features’ layer the RDW-parking locations layer (‘Parkeerlocaties-met-specificaties-RDW-opendata-versie-nov2019_GEO-Parkeergarages_capacity_per_entrance_RD_2-.shp’), resulting in layer ‘Buurt2016_totalparkspaces_TOP10_BGT_2019_BAG2015.shp’.

In the field map of Join Features, the fields BU_CODE, BU_CODE, AANT_INW, BEV_DICHTH, AANTAL_HH, AUTO_TOT, AUTO_HH, AUTO_LAND, BEDR_AUTO, MOTOR_2W, AF_OPRITH, Shape_Area, Cap_x_IO, Parkspace_ (renamed to Pbl_places) and Parkspaces (renamed to Prv_places) were kept and on the latter two (Pbl_places and Prv_places) the Merge Rule ‘Sum’ was applied. This means that for each individual neighborhood this function will sum up the number of parking spaces together.

Next, a field named ‘Tot_places’ was added in which the parking places of Pnl_places and Prv_places are summed with a Field calculator operation

Tot_places = [Pnl_places] + [Prv_places]

Next, the number of parking places per household was calculated:

PP_per_HH = [Tot_places] / [AANTAL_HH]

Next, the number of parking places per hectare was calculated

PP_per_ha = [Tot_places] / ([Shape_Area] / 10000)

Next, a specific metric was calculated that multiplies PP_per_HH with PP_per_ha

PPhhXPPha = [PP_per_HH] * [PP_per_ha]

Next, the total number of registered cars (private and company cars) per neighborhood was calculated

CAR_total = [AUTO_TOT] + [BEDR_AUTO]

Next, the ratio between the number of cars and the number of parking places was calculated

RatioCARPP = [CAR_total] / [Tot_places]

**Variables**

Table 1 provides an overview of variables that are available in the dataset ‘Total_Parkspaces_2019’

**Table 1: Overview of attribute data in ‘Buurt2016_totalparkspaces_TOP10_BGT_2019_BAG2015.shp’**

| **Variable name** | **Description** | **Original dataset** |
| --- | --- | --- |
| BU_CODE | Neighborhood code | Buurt2016 |
| BU_NAME | Neighborhood name | Buurt2016 |
| AANT_INW | Number of inhabitants per neighborhood | Buurt2016 |
| BEV_DICHTH | Population density | Buurt2016 |
| AANTAL_HH | Number of households per neighborhood | Buurt2016 |
| AUTO_TOT | Total number of registered cars per neighborhood | Buurt2016 |
| AUTO_HH | Number of cars per houshold per neighborhood | Buurt2016 |
| AUTO_LAND | Number of cars per km2 | Buurt2016 |
| BEDR_AUTO | Number of delivery vans, trucks, etc. per neighborhood | Buurt2016 |
| MOTOR_2W | Number of motorcycles per neighborhood | Buurt2016 |
| AF_OPRITH | Average distance to motor way exit/entrance | Buurt2016 |
| Shape_Area | Polyon (neighborhood) area | Buurt2016 |
| Cap_x_IO | Total number of parking places RDW | RDW-parking locations layer |
| Pbl_places | Total number of public/paid parking places | Total_parkspaces_2019 |
| Prv_places | Total number of private parking places (with dwellings) | Total_parkspaces_2019 |
| Tot_places | Total number of public/paid and private parking places |  |
| PP_per_HH | Number of parking places per household |  |
| PP_per_ha | Number of parking places per hectare |  |
| PPhhXPPha | Parking places per household * pp per hectare |  |
| CAR_total | Total number of cars and vans per neighborhood |  |
| RatioCARPP | Ratio between number of cars and parking places |  |

**Data provider**

Several

**Data quality**

See description processing. Note that the data has been disaggregated to the neighborhood level but is suitable for disaggregation to the PC6 level or exposure radii around addresses as well.

**Additional information**

n.a.

**Contact information**

GECCO - Geoscience and health cohort consortium

Amsterdam University Medical Centers

Department of Epidemiology and Data Science

Location VUmc, De Boelelaan 1089a, 1081 HV Amsterdam

E: [j.lakerveld@amsterdamumc.nl](mailto:j.lakerveld@amsterdamumc.nl), [a.wagtendonk@amsterdamumc.nl](mailto:a.wagtendonk@vumc.nl)

**Terms and conditions**

None, all public data

**Suggested or required way of data referencing**

GECCO reference

**List of references**

n.a.
