# Supplementary material for: Deep phenotyping meets big data: the Geoscience and hEalth Cohort COnsortium (GECCO) data to enable exposome studies in The Netherlands
Source: Int J Health Geogr. 2020 Nov 13;19:49. doi: 10.1186/s12942-020-00235-z (PMC7662022; doi:10.1186/s12942-020-00235-z)
Supplement: Supplementary file 3 — Additional file 3. Annex S3. [file 12942_2020_235_MOESM3_ESM.docx]

**Land use mix for walkability index 1989 - 2015**

**Spatial coverage** The Netherlands

**Temporal range** 1989, 1993, 1996, 2000, 2003, 2006, 2008, 2010, 2012, 2015

**Data source input data:** CBS Statistics Netherlands – Statistics on Land Use

**Data format input data:** ESRI File Geodatabase (FileGDB) and ESRI Shape files (*.shp)

**Data format output data:** Raster (*.tif) floating point 32 Bit / Point location table (*.csv)

**Data storage output data:** ..\Geodata\ Walkability_indicator\

**Table files (output):** lm89.csv, lm93.csv, lm96.csv, lm00.csv, lm03.csv, lm06.csv, lm08.csv, lm10.csv, lm12.csv, lm15.csv

**GIS files (output):** lmYYYY_DDDDm_z.tif (80 files)

YYYY = 1989, 1993, 1996, 2000, 2003, 2006, 2008, 2010, 2012, 2015

DDDD = 150, 250, 350, 500, 750, 1000, 1650, 2000 meter

**Data description**

The land use mix forms one of the six components in the composite walkability index (see Wagtendonk, Lakerveld, 2019):

1. Population density
2. Density of retail and service destinations (retail environment)
**3. Land-use mix**
4. Street connectivity / intersection density
5. Green space
6. Side walk presence.

Mixed or diverse land use is considered one of the “3Ds”—density, pedestrian friendly design, and diversity—that have been found to associate with walking (Brown et al., 2009). Mixed use brings many diverse walking destinations together in an area, which may be especially important for supporting walking for transportation purposes.

Land-use mix is commonly measured and accepted by means of an entropy index that indicates the heterogeneity of land use. This index is expressed as follows:

$Entropy index=\left( -1 \right)*\frac{[\left( \frac{b1}{a} \right)*\ln\left( \frac{b1}{a} \right)+\left( \frac{b2}{a} \right)*\ln\left( \frac{b2}{a} \right)+etc.]}{ln(n)}$

Where, *a* is the total area in square meter of the different land-uses, and *b* refers to a specific land-use category. Different land-use categories can be included, whereby *b1* refers to category one, *b2* to category two and so on. The variable *n* refers to the total number of land-use categories.^[[1]](#footnote-1)^ The following four land-use categories are often used to constitute the land use mix: 1) Industrial, commercial, public, military and private units, 2) Residential areas, 3) Green urban areas, and 4) Sports and leisure facilities. For the land use mix component in the walkability index we have used the following classes that can be derived from the available land use data from CBS statistics Netherlands:

1. residential
2. commercial
3. social-cultural services
4. offices and public services
5. greenspace and recreation

The land use mix is computed as a z-score for every individual gridcell of 25x25 meters covering the Netherlands using focal statistics (spatial statistics / summaries) for 10 different years and 8 different sized buffers around each raster cell. Resulting grid cell values are linked to all 9 million Dutch address locations and summarized per administrative neighbourhood (1988, 1993, 1995, 1997, 1999, 2001, 2003 – 2018) and PC4 area (1998 – 2017).

**Variable construction**

To build this index we used the land use map series BBG - ‘Bestand BodemGebruik’ which covers the Netherlands for the years 1989, 1993, 1996, 2000, 2003, 2006, 2008, 2010, 2012 and 2015 on a scale of 1:10,000 and geographically corresponds to the topographical map of the Netherlands (TOP10 Nl / TOP10 vektor).

Land use classes change over the years but similar land use categories as suggested for the entropy index are available, such as the following built-up classes: Residential area, industrial area, Retail and catering, Public facilities, Public and socio-cultural facilities and open area classes like Parks and public gardens, sport parks, recreation areas and allotment gardens.

For this index we used the following 5 different land use classes or land use compositions:

| **Land use mix class** | **Classes from BBG** |
| --- | --- |
| 1. residential | woongebied en verblijfsrecreatie (recreatiewoningen) |
| 2. commercial | detailhandel en horeca |
| 3. social-cultural services | sociaal-culturele voorzieningen |
| 4. offices and public services | kantoren en openbare voorzieningen |
| 5. greenspace and recreation | parken en plantsoenen, bos, begraafplaats  sportterreinen en dagrecreatieve terreinen |

As explained, the land use mix is one of six spatial components for the walkability index and is computed as raster cell values in a regular spaced grid covering the Netherlands with raster cells of 25x 25 meter. The individual raster cell values are computed using focal statistics (spatial statistics / summaries) for 10 different years and 8 different sized buffers around each raster cell. In this type of GIS computation a so called ‘moving window analysis’ is applied in which for each raster cell the same calculation (e.g. sum or mean of raster cell values in the window) is carried out that is based on the values of the surrounding raster cells in a buffer of a specific form and size (most often a circular buffer). Figure 1 shows an example of a processing cell with its neighbourhood (left figure) and the concept of the moving analysis window (right figure) where the analysis window moves to the right from processing cell A to B.


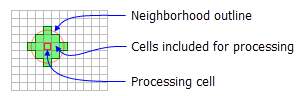

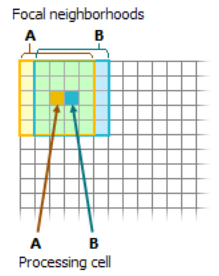


Figure 1 Concept of moving window analysis in focal statistic / neighborhood analysis in GIS

The buffers or neighborhoods concern the size and shape of the area around each raster cell that we consider for walking (the walk ranges), respectively a circular area with a radius of 150-, 250-, 350-, 500-, 750-, 1000-, 1650 and 2000 meter (approximately walking distances between 2 and 30 minutes).

The component values calculated this way for each 25x25 meter location in the Netherlands are extracted and linked to all, approximately 9 million address locations in the Netherlands together with the accompanying XY coordinates. Because this extraction process takes too much time in a conventional ArcGIS-Python script (about an hour for each of the more than 500 produced variables for the walkability index) we have commissioned the company ‘ObjectVision’ to develop a dedicated application on the basis of their ‘GeoDMS’ modelling platform, which is able to produce these 500+ variables in a few hours only.

As well we extract and link these component values to the centroids of all 458.114 PC6 areas (2018) in the Netherlands. Furthermore, statistical summaries are calculated or reported per administrative neighbourhood (1988, 1993, 1995, 1997, 1999, 2001, 2003 – 2018) and PC4 area (1998 – 2017).

The spatial components that are input for the walkability index on each location were created in a Geographical Information System (GIS software: ArcGIS desktop 10.6/7 from ESRI) involving spatial data processing and analysis of raster and vector map layers. Because these GIS operations have to be repeated for a large number of years and walk ranges we automated them using Python scripts that apply the ArcPy modules of ArcGIS.

**GIS data processing**

A summary of GIS operations carried out to produce the required table variables is presented here below. For exact procedures we refer to the Python scripts used to automate these GIS operations.

| **Adress level**  *Python script: 3_Land_use_mix_Walkability.py*   - Convert vector to raster (25 meter cells) - Reclassify land use maps to suggested classes (as far as possible) - Calculate focal statistics (summed area) per year, combined land use classes and exposure area (component a) - Calculate focal statistics (summed area) per year, land use class and exposure area (components b1, b2, b3 and b4) - Use raster calculation to calculate entropy index for each gridcell - Calculate z-score for each raster cell (raster cell value - Mean) / SD)   *Geo-dms application with adapted lines in ‘stam.dms’*   - Extract values to address coordinate table for each exposure variable   **PC6 level**  *Python script:* *3_Land_use_mix_extract_values_2_PC6.py*   - Extract values to PC6 centroid table for each exposure variable   *Geo-dms application with adapted lines in ‘stam.dms’*   - Extract values to address coordinate table for each exposure variable   **PC4/Neighborhood level**  *Python script: 3_Land_use_mix_24jul19_administrative_units.py*   - Calculate zonal statistics per neighbourhood and PC-4 area and use raster calculation to calculate entropy index for neighbourhood / PC-4 area |
| --- |

**Map example z-scores for entropy index / land use mix 2015 - 1000 meter radius**C:\Work\VUmc-GECCO\Geodata\Walkability_indicator\Map overview walkability components.mxd

| 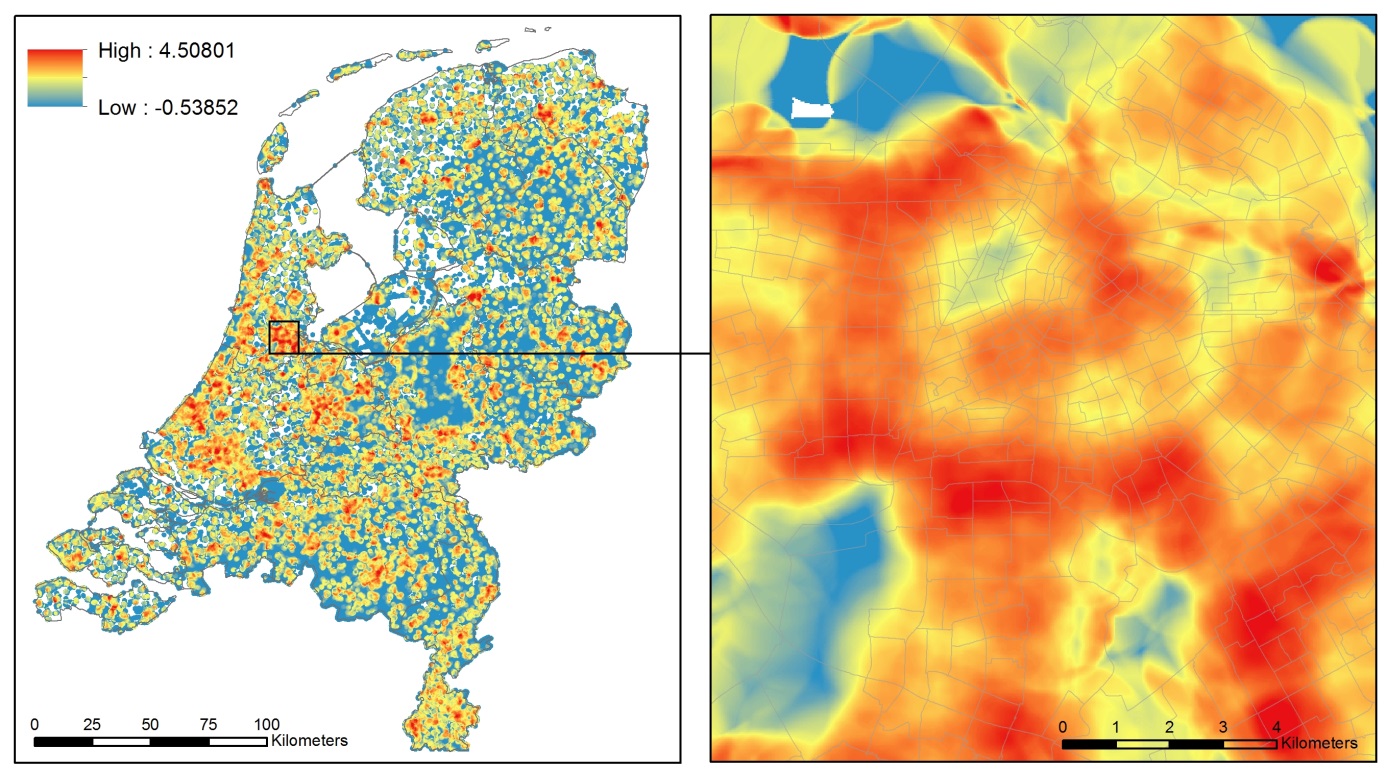 |
| --- |

**Data corrections**

Before we could operationalize the steps described above in GIS, we needed to deal with two specific issues.

The **first issue** concerns a problematic land use class namely class number 4 ‘industry and offices’. This class is represented as the land use class business park (‘bedrijfsterreinen’) in the CBS bodemgebruikbestanden and concerns a mixed class that can represent industrial plants, business parks (trade / manufacturing, etc.) and office space. Following Brown et al. (2009), we assume that the presence of industrial terrains and business parks does not contribute to the walkability of a neighborhood, while on the contrary, offices can be considered walking destinations that contribute to utilitarian walking. This line of thought is also supported by Duncan et al. (2010) who demonstrate that significant relations between land use heterogeneity and walking behavior can only be expected when land uses with limited relevance for the type of walking studied are excluded. For this reason we decided to exclude industrial land use from our land-use mix and split up the class ‘business park’ to the classes ‘business park and industrial plants’ and the class ‘office space’ using the attribute field ‘Gebruiksdoel’ (= use objective) in the dataset Basisregistratie Adressen en Gebouwen (BAG) for the disaggregation process. In this attribute field office functions are distinguished from functions for industrial processing/storage of materials or agricultural purposes. The BAG dataset we use concerns the year 2015. This means that for a certain land use mix year, e.g. 2012, it can happen that the use objective in BAG in 2015 is different from the use objective in 2012, but we assume the number of function changes between office space and business or industrial space will be relatively small.

More details about this disaggregation process can be found in the appendix.

The **second issue** concerns the land use class ‘commercial’. Because CBS uses detailed retail data from Locatus to enrich the class ‘commercial’ (‘detailhandel’ and ‘horeca’) from 2008 onwards (personal information Hans Visser / CBS, see e-mail d.d. 5 juni 2019), land use data before that year is much less detailed for the class commercial.

To correct for these differences, we made the following assumptions:

- established retail areas do not change often (and are part of municipal zoning plans)
- the majority of new retail areas are established in urban expansion areas

On the basis of these assumptions we took the retail area on the BBG map of 2008 and used the CBS maps ‘bevolkingskernen’ (showing morphological continuous areas that are mainly inhabited by people) available for the years 2001, 2008 and 2011 to determine the urban expansion areas between 2001 and 2008. Subsequently, we used these areas to remove the more recent retail area in the BBG land use maps of 2000 and 2003. Before 2001 there are no CBS maps ‘bevolkingskernen’ available, but the Dutch [environmental assessment agency](https://context.reverso.net/vertaling/engels-nederlands/environmental+assessment+agency+%28Planbureau+voor+de+Leefomgeving) (PBL) has produced generalized urbanization maps for several years (among others 1980, 1989 and 1993) which we used in a similar manner to correct the land use maps of 1989 and 1993. The following overview shows the corrections made for each land use map.

- BBG land use 1989: retail class corrected with urban expansion 1989 – 2008
- BBG land use 1993: retail class corrected with urban expansion 1993 – 2008
- BBG land use 1996: retail class corrected with urban expansion 1993 – 2008
- BBG land use 2000: retail class corrected with urban expansion 2001 – 2008
- BBG land use 2003: retail class corrected with urban expansion 2001 – 2008
- BBG land use 2006: retail class corrected with retail in BBG land use map 2008
- BBG land use 2008 and onwards: no retail correction necessary

**Variables**

**Table 1:** overview of variables in GIS datasets (*.tif)

| **Variable name** | **Description** |
| --- | --- |
| **Raster value** | Z-score land use mix per 25x25 grid cell ranging between ca. -1 and 17 |

**Table 2:** overview of variables in table datasets (*.csv)

| **Variable name** | **Description** |
| --- | --- |
| **postcode** | Z-score land use mix per 25x25 grid cell ranging between ca. -1 and 17 |
| **huisnummer** | House number |
| **huisletter** | House letter |
| **toevoeging** | House number / letter addition |
| **nummeraanduiding_id** | Number indication id |
| **X** | X coordinates Dutch coord. system (Rijksdriehoek-stelsel) |
| **Y** | Y coordinates Dutch coord. system (Rijksdriehoek-stelsel) |
| **z150m** | z-score for 150 meter radius |
| **z250m** | z-score for 250 meter radius |
| **z350m** | z-score for 350 meter radius |
| **z500m** | z-score for 500 meter radius |
| **z750m** | z-score for 750 meter radius |
| **z1000m** | z-score for 1000 meter radius |
| **z1650m** | z-score for 1650 meter radius |
| **z2000m** | z-score for 2000 meter radius |

**Discussion**

We considered to use a remaining class to prevent that the land use mix score becomes zero in e.g. a mainly forested area. However, adding all other land uses resulted in negative entropy scores and strictly speaking it makes sense that the diversity in the land use mix becomes zero when only one land use class is present. In the case of forest (green space) this will also be a separate component in the walkability score. Moreover, adding other classes would mean they will contribute to the heterogeneity of the land use while they are considered not relevant for peoples walking behavior (see further below). Therefore, we did not use a class with remaining land use categories.

Another point of concern is that according to Hajna et al. (2014) and also brought forward by Brown et al. (2009), the entropy index has a major limitation which should be addressed, because entropy remains constant when distinct land-use types remain in constant relative proportions; yet mixing or integration of land uses can change dramatically. For example for the entropy index is does not make any difference if the proportions between residential and commercial land use are 80/20 or 20/80, while this makes a big difference for walking behavior, e.g. for the amount of transport walking. Im and Choi (2018) made this effect clear in a figure, see Figure , and named it the ‘n-shaped characteristic’ of the LUM (Land-Use Mix) index, but do not offer other alternatives for the LUM index than recommendations for its proper use.


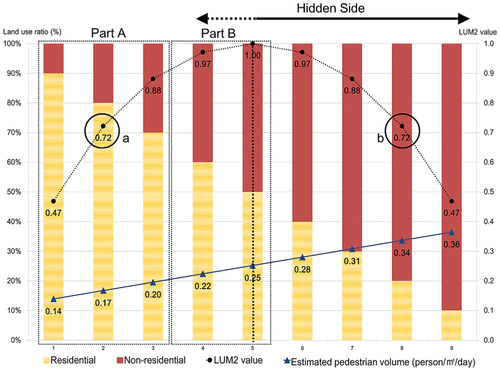


Figure 2 The ‘n-shaped characteristic’ of the LUM (Land-Use Mix) index according to Im and Choi (2018)

If we inspect our resulting land use mix maps, we see indeed different unexpected patterns that relate probably to the problem described here above. In the map example in Figure that displays the land use mix entropy for a walk range of 1000 meter radius for the municipality of Amersfoort, we see among others high unexpected entropy values in the northwest part of the map (indicated with the blue circle) around an area dominated by industry and company sites. These effects are particularly visible in the maps based on large buffer radii and therefore we recommend to improve this component and maybe avoid to use buffer sizes larger than e.g. 750 meter.


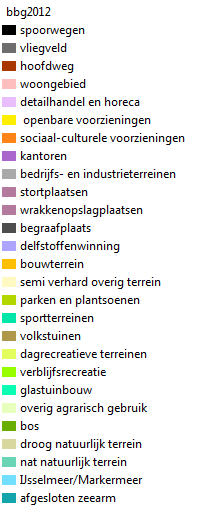

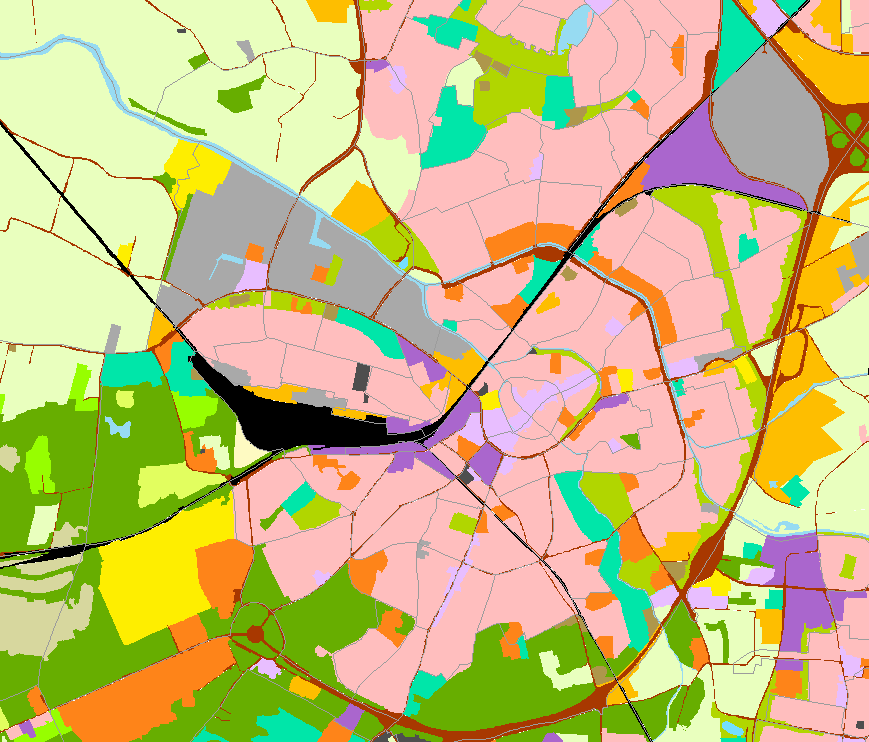

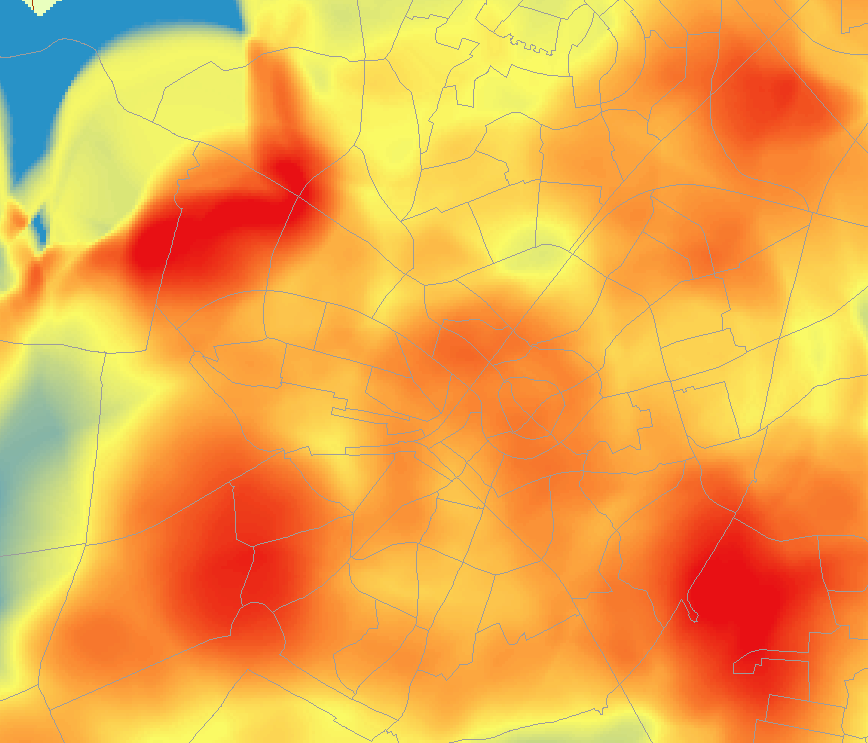


Figure 3 Map example entropy index land use mix, with 1000 meter radius walk range (municipality of Amersfoort)

**Source**

The land use map series BBG - ‘Bestand BodemGebruik’ for the years 1989, 1993, 1996, 2000, 2003, 2006, 2008, 2010, 2012 and 2015 is produced by CBS statistics Netherlands. The files for 1989 and 1993 have been acquired by specific request to CBS (Hans Visser: [j.visser@cbs.nl](mailto:j.visser@cbs.nl)). Source data for the years 1996, 2000, 2003 and 2006 was acquired from DANS-KNAW (Data Archiving and Networked Services). Source data for the years 2008 to 2015 was downloaded from:
<http://geodata.nationaalgeoregister.nl/bestandbodemgebruik2008/extract/bestandbodemgebruik2008.zip>
<http://geodata.nationaalgeoregister.nl/bestandbodemgebruik2010/extract/bestandbodemgebruik2010.zip>

All available files are in vector format.

**Data quality**

See information provided by CBS statistics Netherlands in ‘Productbeschrijving Bestand Bodemgebruik’ (2008, 2015)

**Contact information**

Statistics Netherlands – Statistics on Land Use [In Dutch: Centraal Bureau voor de Statistiek – Bodemstatistiek].

Contactpersoon: Hans Visser (Team Regio & Ruimte)

E-mail: [j.visser@cbs.nl](mailto:j.visser@cbs.nl)

Adres: Henri Faasdreef 312, Postbus 24500, Den Haag, Zuid Holland, 2490HA, Nederland

E-mail: [infoservice@cbs.nl](mailto:infoservice@cbs.nl)

Telephone: 0031 (0)88 570 70 70

Processed data Gecco

Alfred J. Wagtendonk

Department of Epidemiology and Biostatistics

Location VUmc, De Boelelaan 1089a, 1081 HV Amsterdam

E-mail: a.wagtendonk@amsterdamumc.nl

**Terms and conditions**

Citation obliged.

Licenses: <http://creativecommons.org/licenses/by/4.0/nl/>

**List of references**

Brown, B. B., Yamada, I., Smith, K. R., Zick, C. D., Kowaleski-Jones, L., & Fan, J. X. (2009). Mixed land use and walkability: Variations in land use measures and relationships with BMI, overweight, and obesity. *Health & place*, *15*(4), 1130-1141.

CBS (2008, 2015). Bestand bodemgebruik – productbeschrijving. Centraal Bureau voor de Statistiek, Den Haag

Duncan, M. J., Winkler, E., Sugiyama, T., Cerin, E., duToit, L., Leslie, E., & Owen, N. (2010). Relationships of land use mix with walking for transport: do land uses and geographical scale matter? *Journal of urban health: bulletin of the New York Academy of Medicine*, *87*(5), 782–795.

Hajna, S., Dasgupta, K., Joseph, L., & Ross, N. A. (2014). A call for caution and transparency in the calculation of land use mix: measurement bias in the estimation of associations between land use mix and physical activity. *Health & place*, *29*, 79-83.

Im, H. N., & Choi, C. G. (2018). The hidden side of the entropy-based land-use mix index: Clarifying the relationship between pedestrian volume and land-use mix. *Urban Studies*, 0042098018763319.

Wagtendonk, A.J., Lakerveld, J. (2019) Walkability score Netherlands version 1.0 - Technical document, Available via <https://www.gecco.nl>

**APPENDIX:** Steps for dissaggregation land use class business park CBS Bodemgebruik

The following steps were carried out to disaggregate the land use class business park CBS Bodemgebruik to classes ‘business park and industrial plants’ and the class ‘office space’ using attribute field ‘Gebruiksdoel’ in BAG.

1. Select points with Gebruiksdoel Industriefunctie from BAG point layer ‘verblijfsobject’ (other mixed function groups were not selected)
2. Use (1) to Select by location aunits from the BAG layer ‘Pand’ that intersect with 1.
3. Export selected polygons to new layer named ‘Industry2015_BAG_BBG2012.shp’. Add field named ‘Industrie’
4. Repeat 1 to 3 for Gebruiksdoel Kantoorfunctie and export to layer ‘Kantoor2015_BAG_BBG2012.shp’. Add field named ‘Kantoor’
5. Select class ‘bedrijfsterreinen’ from BBG and export to separate polygon layer named ‘Bedrijfsterreinen_2012.shp’.
6. Carry out spatial join (intersect) between ‘Bedrijfsterreinen_2012’ (target layer) and ‘Kantoor2015_BAG_BBG2012.shp’ (join layer). Make sure you set a merge rule on the field ‘SHAPE_AR_1’: SUM. This takes care all that the areas in m2 of all the kantoor units that are joined to a certain polygon in Bedrijfsterreinen_2012 are added to a total area per polygon. Name result: ‘BBG2012_SpatJoinBAG2015_kantoor.shp’
7. Next carry out spatial join (intersect) between ‘BBG2012_SpatJoinBAG2015_kantoor.shp’ (target layer) and ‘Industry2015_BAG_BBG2012.shp’ (join layer). Make sure you set a merge rule on the field ‘SHAPE_AR_2’: SUM. This takes care all that the areas in m2 of all the industry units that are joined to a certain polygon in BBG2012_SpatJoinBAG2015_kantoor.shp are added to a total area per polygon. Name result: ‘BBG2012_SpatJoinBAG2015_kantoor_industry.shp’.
8. Now add a new field (integer short) to ‘BBG2012_SpatJoinBAG2015_kantoor_industry.shp’ named ‘Type_new’. Carry out the following attribute selections:

SELECT FROM ‘BBG2012_SpatJoinBAG2015_kantoor_industry.shp’ WHERE:
"SHAPE_Ar_1" > "SHAPE_Ar_2" (total office space is larger than total industrial space). Now use the Field calculator to give a value of 1 to the selected cells.
SELECT FROM ‘BBG2012_SpatJoinBAG2015_kantoor_industry.shp’ WHERE:
"SHAPE_Ar_2" >= "SHAPE_Ar_1" (total industrial space is larger than total office space). Now use the Field calculator to give a value of 2 to the selected cells.
SELECT FROM ‘BBG2012_SpatJoinBAG2015_kantoor_industry.shp’ WHERE:
"SHAPE_Ar_1" = "SHAPE_Ar_2" (total industrial space is equal to total office space, in most cases these concern polygon without values (0 m2) for office and industrial space). Now use the Field calculator to give a value of 0 to the selected cells.

1. Export only the new field ‘Type_new’ to a separate layer (name it e.g. ‘Type_new.shp’) and select only ‘Kantoor polygons’ and use this layer to carry out a spatial selection (Select by location / ‘Are identical to the source layer feature’) in BBG2012. Add a new field to the BBG2012 layer named ‘BG2012Updt’ and assign the value 25 for office space to the selected records. Switch the selection and give all other records their original value from the field ‘BG2012A’.

1. Bahadure, S. & Kotharkar, R. Assessing Sustainability of Mixed Use Neighbourhoods through Residents’ Travel Behaviour and Perception: The Case of Nagpur, India. *Sustainability*. 2015. [↑](#footnote-ref-1)
